# Supplementary material for: Integrative and comparative genomic analyses identify clinically relevant pulmonary carcinoid groups and unveil the supra-carcinoids
Source: Nat Commun. 2019 Aug 20;10:3407. doi: 10.1038/s41467-019-11276-9 (PMC6702229; doi:10.1038/s41467-019-11276-9)
Supplement: Supplementary file 1 — Supplementary Information [file 41467_2019_11276_MOESM1_ESM.pdf]

Supplementary Information
Integrative and comparative genomic analyses identify clinically relevant groups of pulmonary carcinoids and unveil the supra-carcinoids

Alcala *et al.*

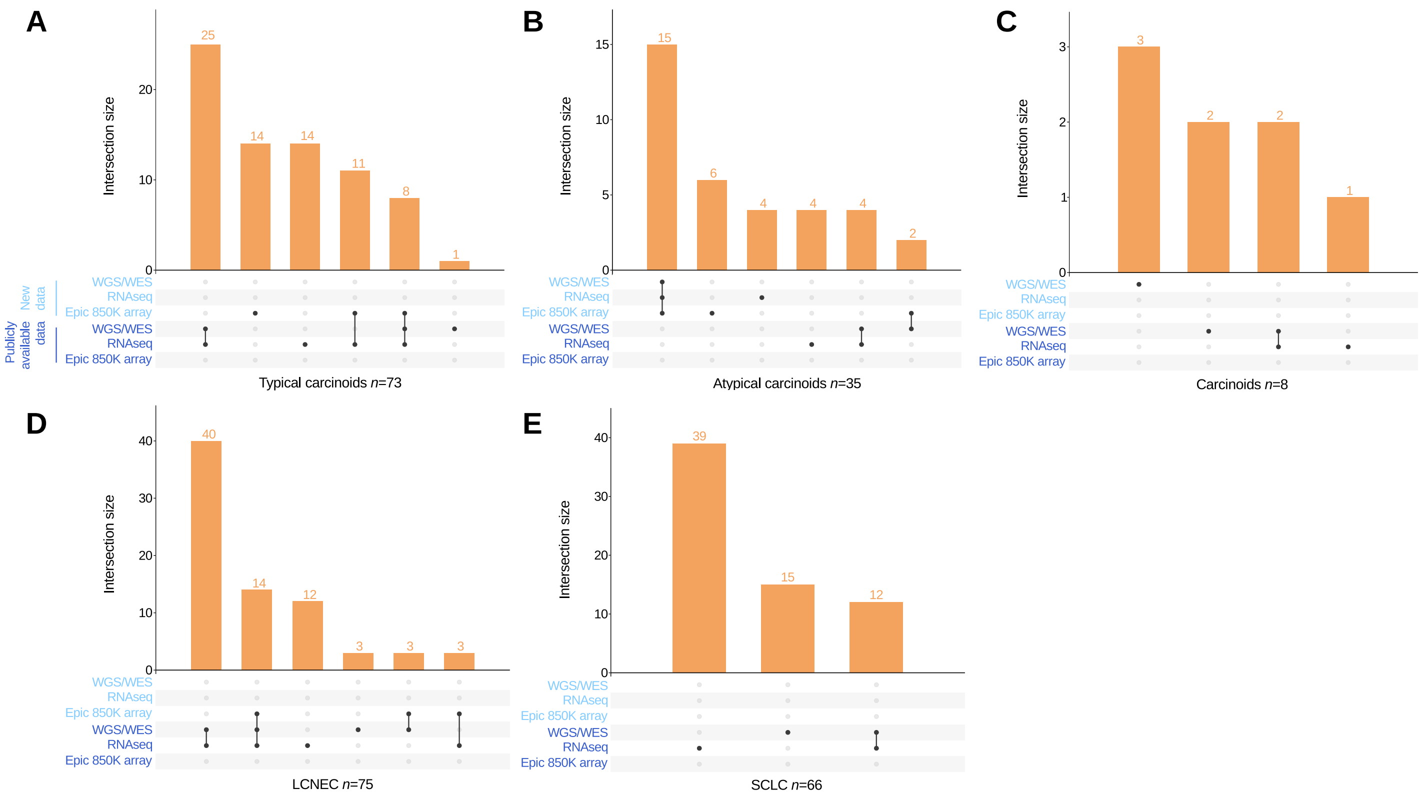

Supplementary Figure 1 Overview of the multi-omic experimental design for LNEN samples. Overview of the number of samples with whole-genome sequencing (WGS) or whole-exome sequencing (WES), RNA-sequencing (RNA-seq), and Epic 850K methylation arrays (EPIC 850K array), for (A) typical carcinoids, (B) atypical carcinoids, (C) carcinoids, (D) large cell neuroendocrine carcinoma (LCNEC), and (E) small cell lung cancer (SCLC). In all panels, new (light blue) and publicly available (dark blue) data are mentioned separately. The total number of samples (n) are indicated next to each cancer type. Data necessary to reproduce the figure are provided in Supplementary Data 1.

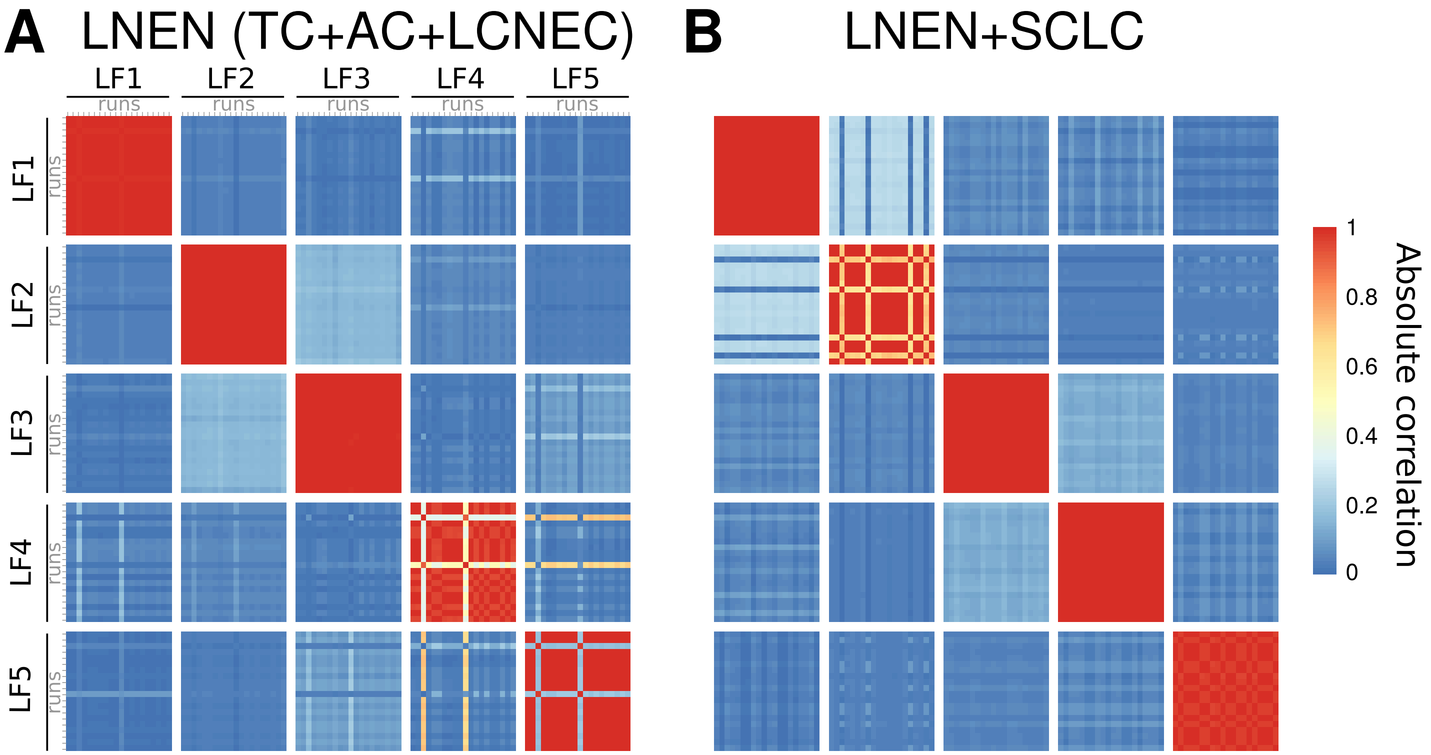

Supplementary Figure 2 Robustness of the MOFA latent factors presented in Figure 1A. Each panel corresponds to the matrix of Pearson correlation coefficients between latent factors (LFs) from 20 replicate MOFA runs. Rows/columns correspond to a single LF from a single MOFA run; rows/columns are clustered by LF (from 1 to 5), and ordered by run number (from 1 to 20) within a cluster (100 row/column in total). Colours represent the strength of the absolute correlation (red for high correlation, blue for low correlation). A) Correlation between LF across runs for MOFA run on all LNEN samples (the best run among the 20 is presented Figure 1A and Supplementary Figure 13B). B) Correlation between LF across runs for MOFA run on all LNEN and SCLC samples (the best run among the 20 is presented Supplementary Figure 13A). In all panels, the red colour on the diagonal and the blue colours off-diagonal indicate a very good robustness of the LF. Data necessary to reproduce the figure are provided in Supplementary Data 1.

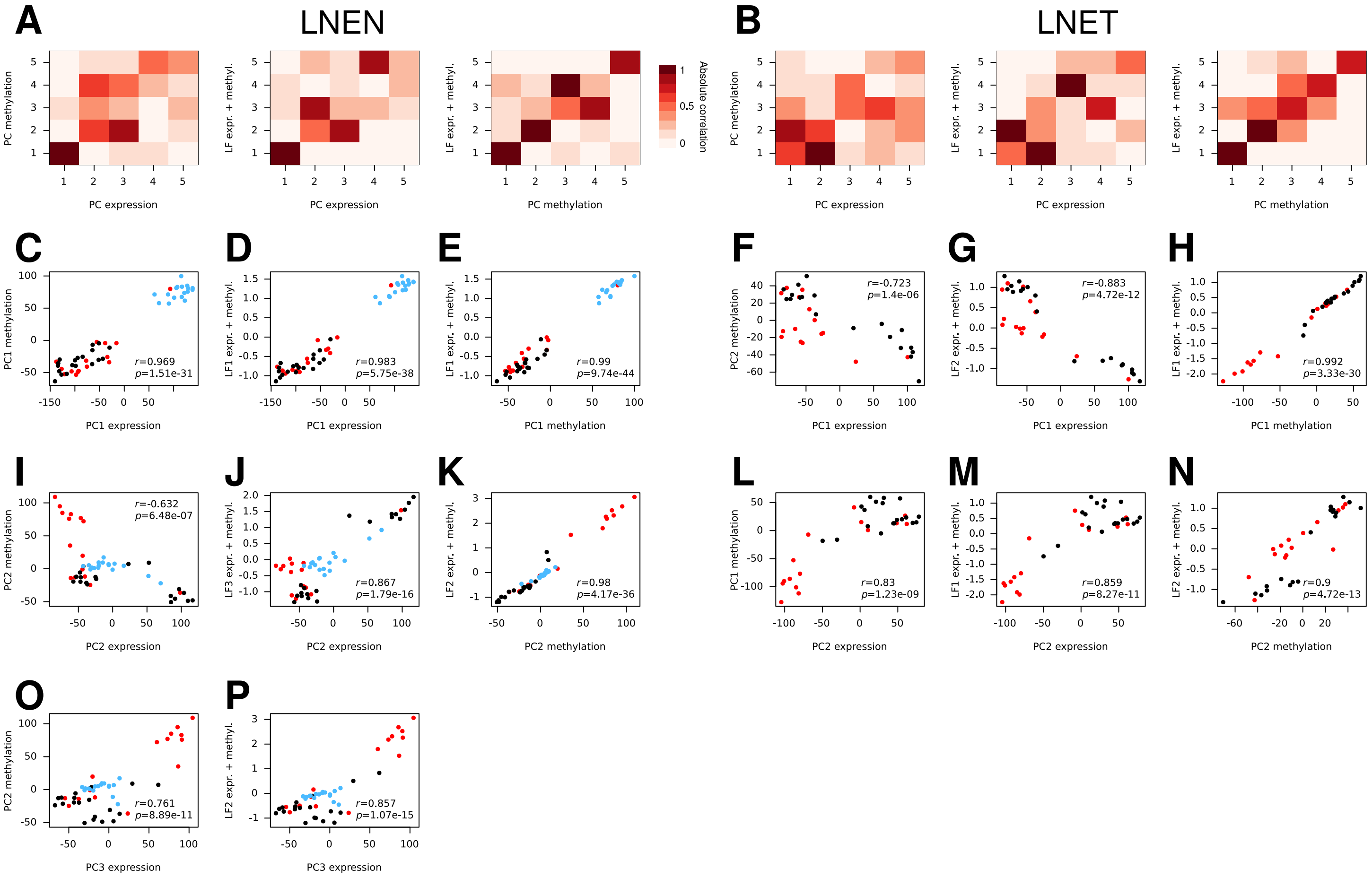

Supplementary Figure 3 Correlations between MOFA latent factors (Figures 1A and 4A) and the principal components of the PCA of expression (Supplementary Figure 6) and methylation (Supplementary Figure 7). Panels (A) and (B) present the correlation matrices between expression and methylation PCA (left), between expression PCA and MOFA (middle), and between methylation PCA and MOFA (right), for MOFA on LNEN samples and LNET samples, respectively. Panels (C)-(P) highlight the strongest correlations from panels (A) and (B) in the form of scatter plots, and display Pearson correlation coefficients and the p-values of the associated tests. Atypical, Typical and LCNEC samples are represented in red, black and blue respectively. Data necessary to reproduce the figure are provided in Supplementary Data 1, 2 and 3.

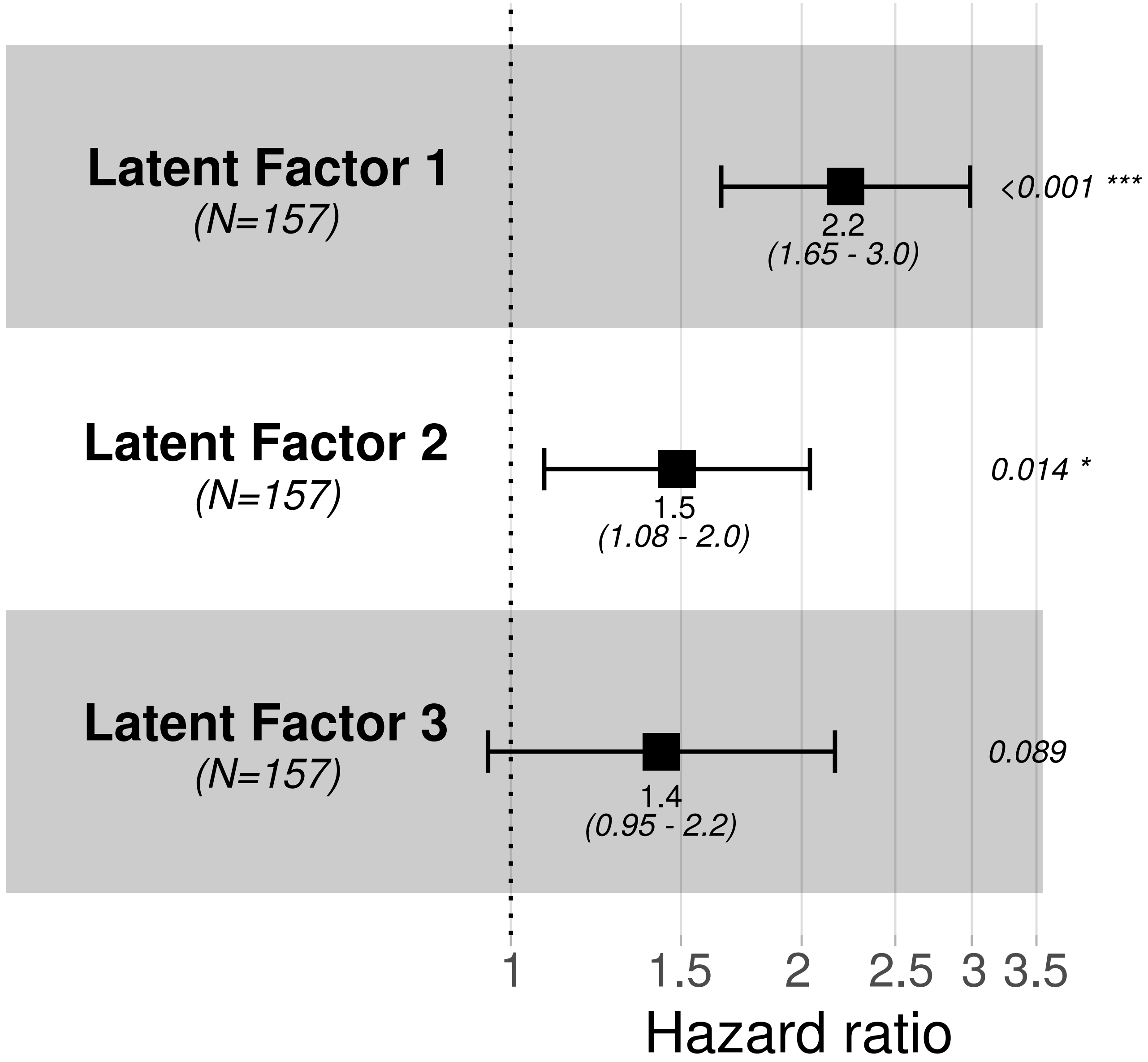

Supplementary Figure 4 Forest plot of the survival analysis based on the first three MOFA latent factors (LFs) of LNEN samples from Figure 1A. Results correspond to a Cox proportional hazards model with coordinates of samples on the first 3 MOFA LFs as continuous explanatory variables. The black box represents estimated hazard ratios and whiskers represent the associated 95% confidence intervals. Wald test p-values are shown on the right; $0.01 \leq p < 0.05$, $0.001 \leq p < 0.01$, and $p < 0.001$ are annotated by one, two, and three stars, respectively. Number of samples (N) for each group is given in brackets. Data necessary to reproduce the figure are provided in Supplementary Data 1.

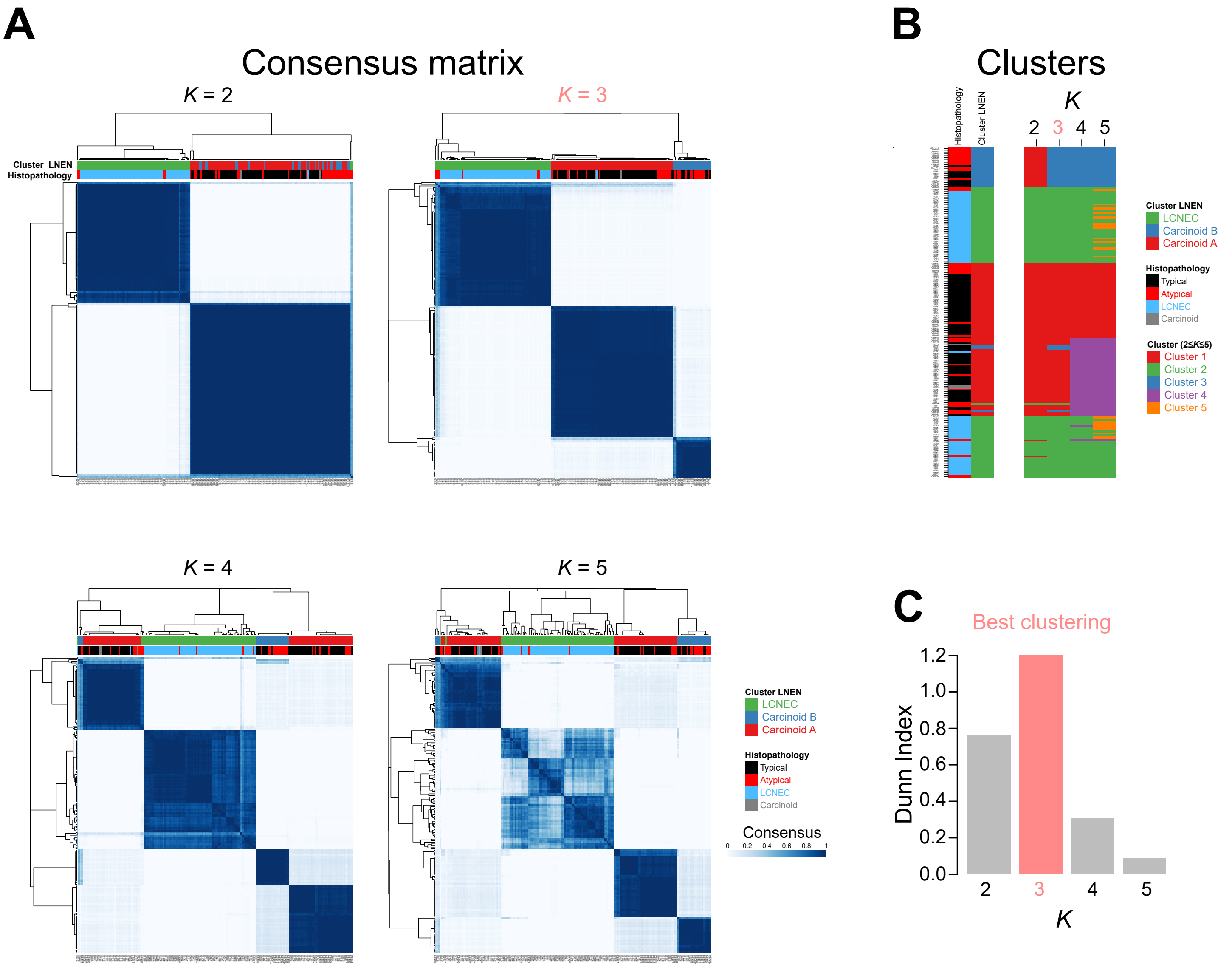

Supplementary Figure 5 Robustness of the consensus clustering of LNENs presented in Figure 1A. A) Heatmap of the consensus matrix for four numbers of clusters *K*; cluster memberships and histopathological types are reported above the columns, and the dendrogram represents a hierarchical clustering. B) Cluster membership as a function of *K*. C) Clustering quality metric (Dunn Index) for each value of *K*; the best clustering according to the metric is highlighted in pink. Data necessary to reproduce the figure are provided in Supplementary Data 1.

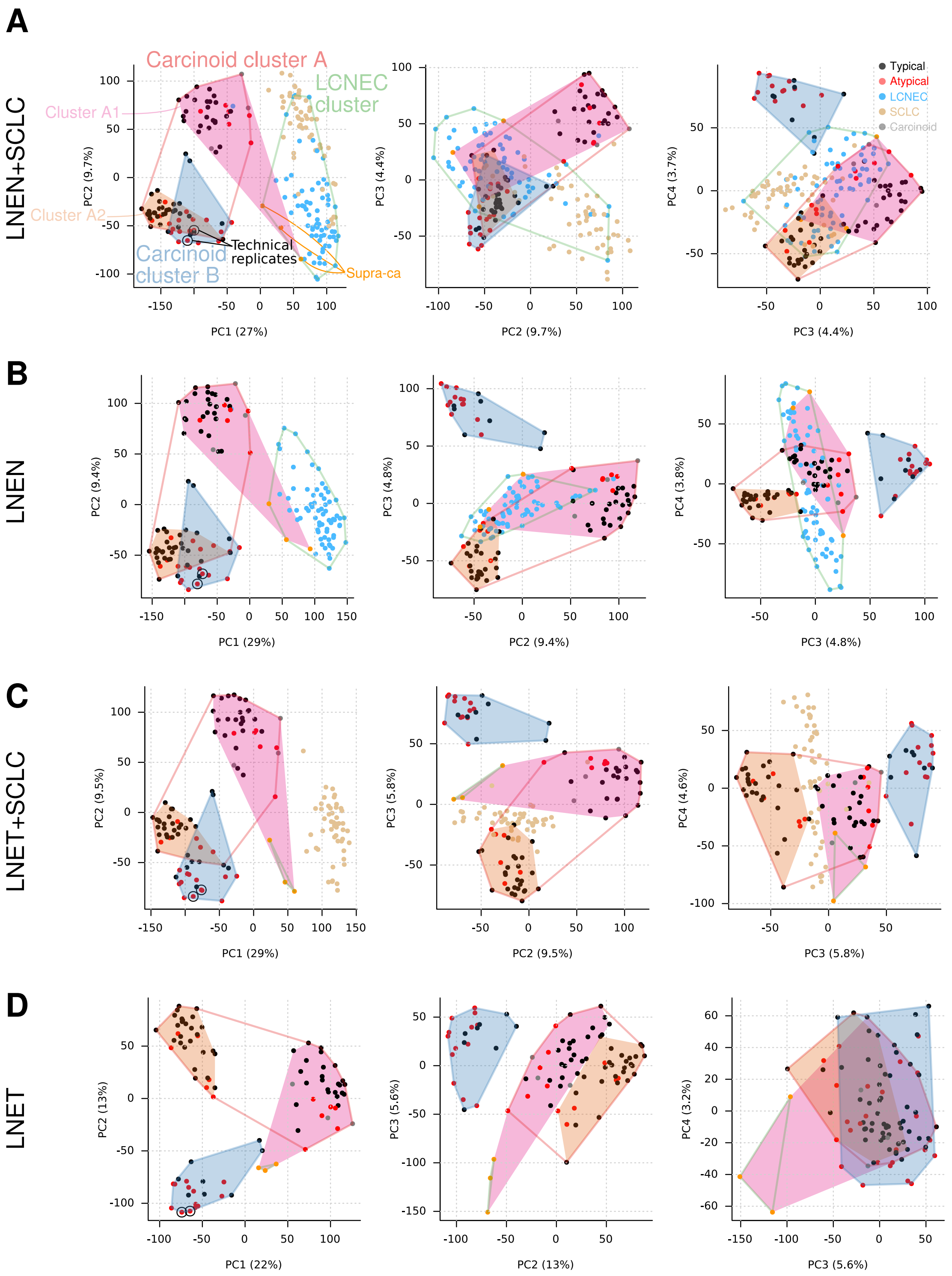

Supplementary Figure 6 Principal Component Analysis (PCA) of transcriptome data. A) PCA of transcriptomes of typical and atypical carcinoids, LCNEC (i.e., LNEN), and SCLC. B) PCA of transcriptomes of typical, atypical carcinoids, and LCNEC (i.e., LNEN). C) PCA of transcriptomes of typical, atypical carcinoids (i.e., LNET), and SCLC. D) PCA of transcriptomes of typical and atypical carcinoids (i.e., LNET). On each panel, point colors correspond to histopathological types (black for typical, red for atypical, grey for carcinoids, blue for LCNEC, beige for SCLC) and supra-carcinoids (orange), polygons correspond to the LNEN clusters from Figure 1A, and filled surfaces correspond to LNET clusters from Figure 4A; their shapes correspond to the convex hull of samples from the focal cluster. The two technical replicates are circled in black. Data necessary to reproduce the figure are provided in Supplementary Data 2.

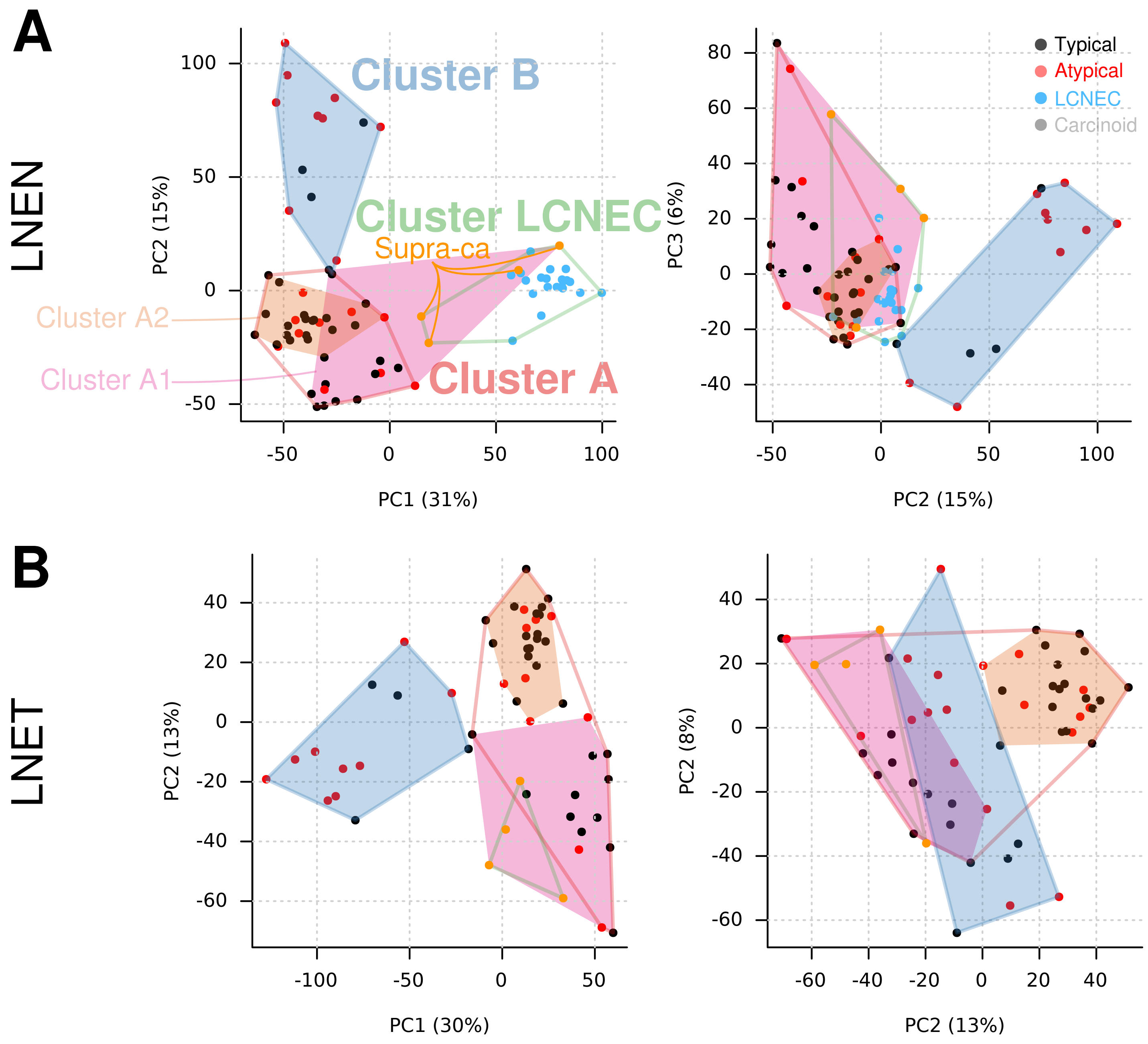

Supplementary Figure 7 Principal Component Analysis (PCA) of the methylation data. A) Analysis of all samples (LNEN). B) Analysis restricted to LNET samples. Figure design follows that of Supplementary Figure 6. Data necessary to reproduce the figure are provided in Supplementary Data 3.

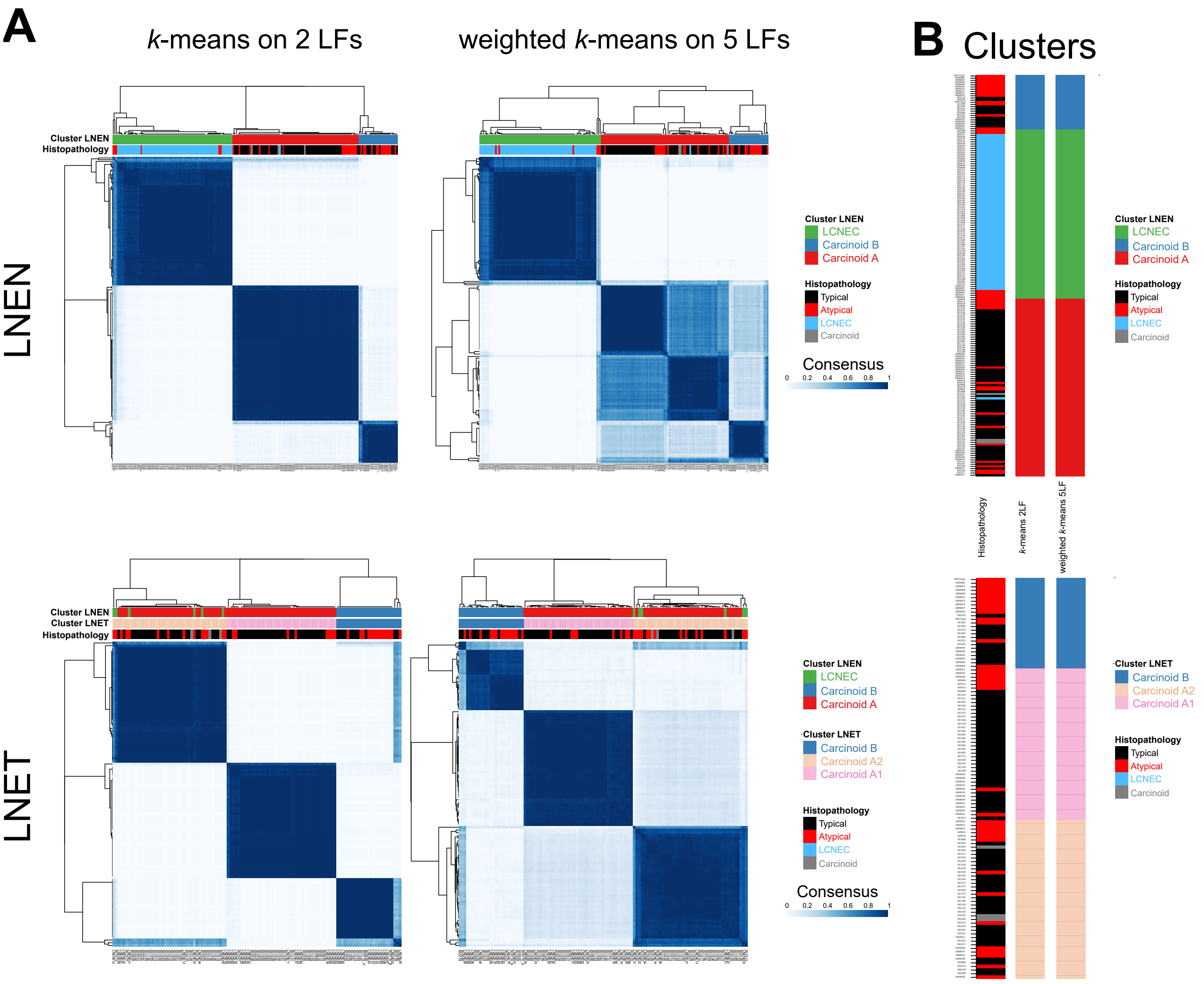

Supplementary Figure 8 Comparison between consensus clustering on MOFA latent factors based on different clustering algorithms. A) First column: copied from Supplementary Figures 5A and 18A; *k*-means clustering using the first 2 latent factors, for LNEN (top) and LNET (bottom) samples. Second column: weighted *k*-means clustering using the 5 latent factors identified by MOFA, weighted by their proportion of variance explained. B) Histopathological type (first column) and cluster membership of each sample, for consensus clustering using the *k*-means algorithm on the first 2 latent factors (second column), and using the weighted *k*-means algorithm on all 5 latent factors (third column). Data necessary to reproduce the figure are provided in Supplementary Data 1.

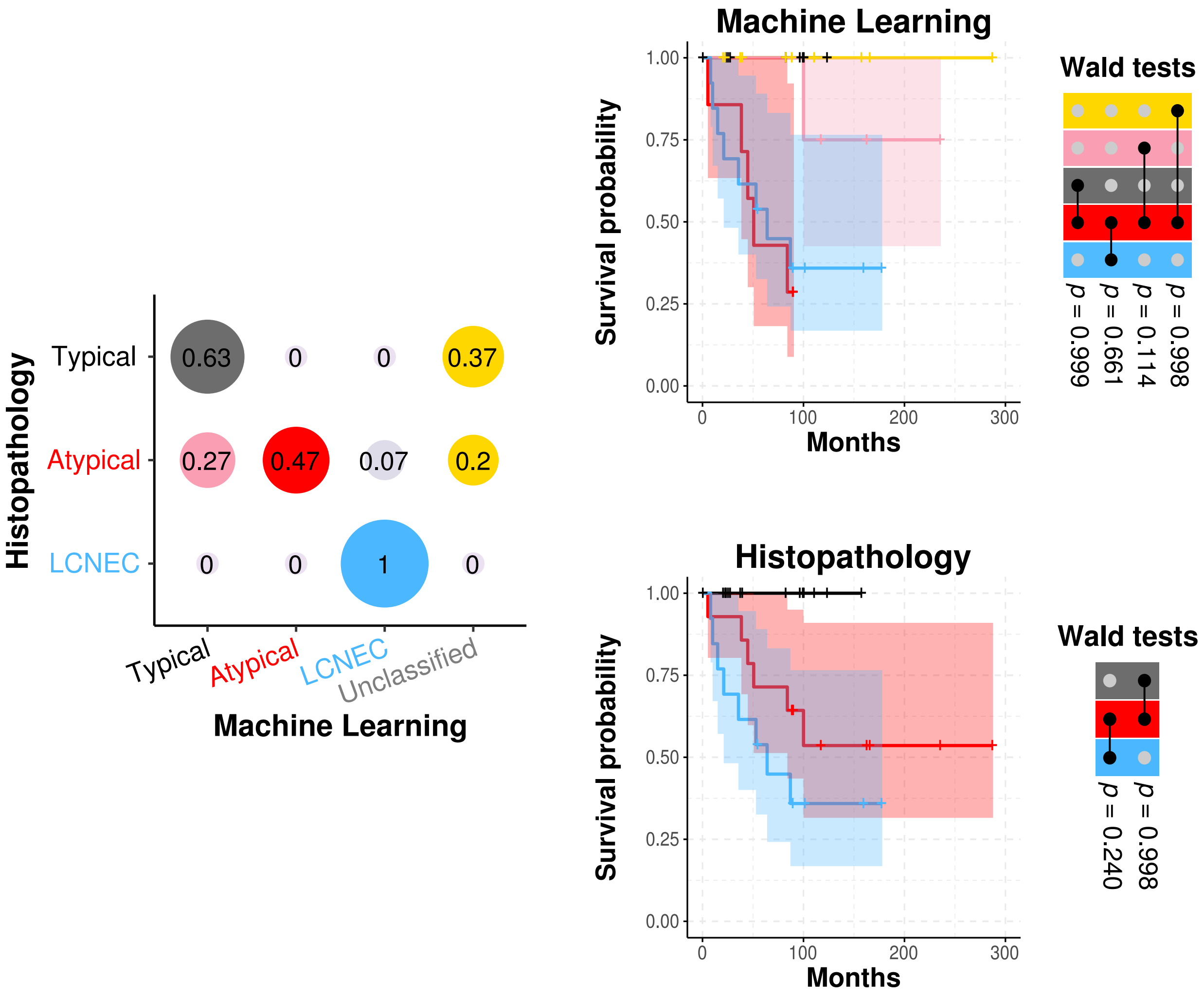

Supplementary Figure 9 Analysis of the ML predictions based on a model integrating expression and methylation data simultaneously. The analysis is similar to that used to produce Figure 1B-C, except that expression and methylation data are integrated simultaneously in the model rather than independently (see Online Methods). Figure design follows that of Figure 1B-C. Data necessary to reproduce the figure are provided in Supplementary Data 1.

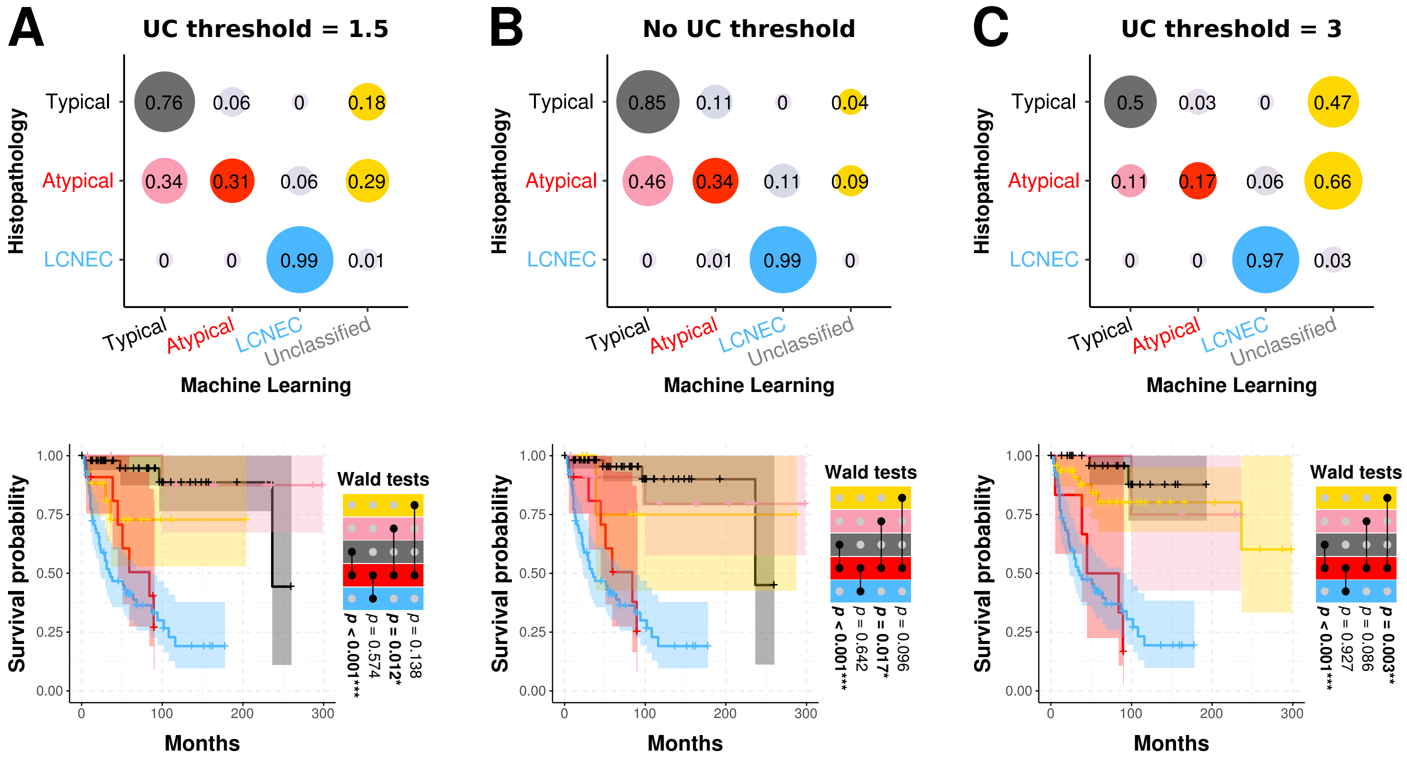

Supplementary Figure 10 Comparison of the ML predictions when applying different thresholds to define the "Unclassified" category. A) Copied from Figure 1B-C for reference. Upper panel : Confusion matrix associated with the ML predictions combined using expression and methylation-based predictions (see Online methods) and a threshold of 1.5 for the definition of the "Unclassified" category. Lower panel: Kaplan-Meier curves of the overall survival of the different ML-predictions groups. B) Upper panel: Confusion matrix associated with the ML predictions combined using expression and methylation-based predictions and no threshold for the definition of the "Unclassified" category. In this case, the only samples predicted as "Unclassified" are the ones with discordant expression-based and methylation-based predictions. Lower panel: Kaplan-Meier curves of the overall survival of the different ML-predictions groups. C) Upper panel: Confusion matrix associated with the ML predictions combined using expression and methylation-based predictions and a threshold of 3 for the definition of the "Unclassified" category. Lower panel: Kaplan-Meier curves of the overall survival of the different ML-predictions groups. For each Kaplan-Meier plot, the colour associated to each group matches that of the confusion matrix in the upper panel. Next to each Kaplan-Meier plot, matrix layouts represent pairwise Wald tests between the reference group (in red) and the other groups, and the associated p-values; $0.01 \leq p < 0.05$, $0.001 \leq p < 0.01$, and $p < 0.001$ are annotated by one, two, and three stars, respectively. Data necessary to reproduce the figure are provided in Supplementary Data 1.

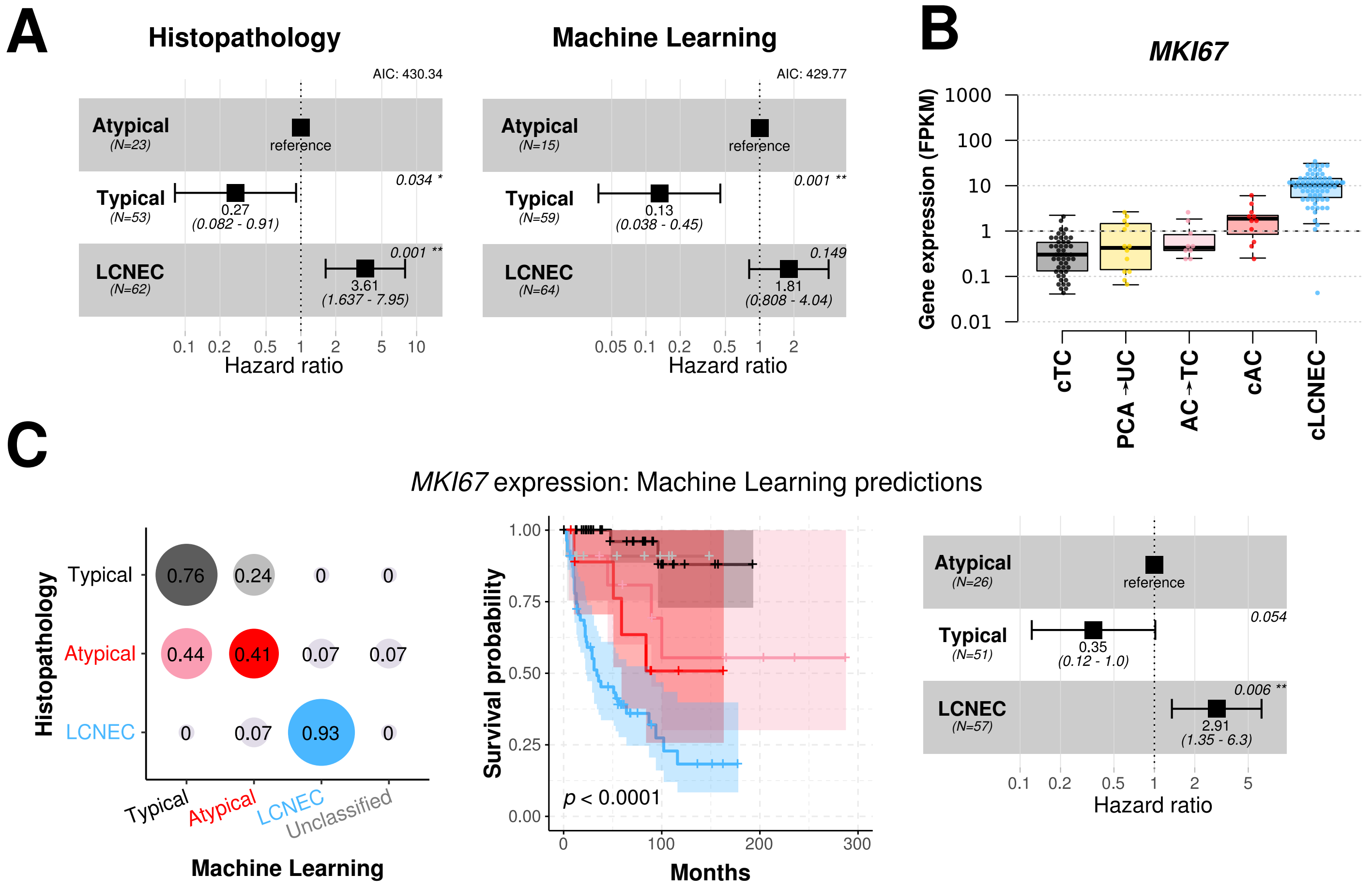

Supplementary Figure 11 Comparison of overall survival based on different classifications. A) Forest plot of hazard ratios of overall survival for two alternative models. Left panel: a model based on the histopathological report. Right panel: a model based on the machine learning predictions from expression and methylation data. For the two models, the same set of 138 samples was considered (see Online methods). B) Boxplot of the expression level (in Fragments Per Kilobase Million; FPKM) of *MKI67* for each prediction group highlighted in Figure 1B. cTC (consensus typical) are typical samples predicted as typical, PCA->UC carcinoids predicted as unclassified, AC->TC atypical samples predicted as typical, cAC (consensus atypical) atypical samples predicted as atypical and cLCNEC (consensus LCNEC) LCNEC samples predicted as LCNEC. Centre line represents the median and box bounds represent the inter-quartile range (IQR). The whiskers span a 1.5-fold IQR or the highest and lowest observation values if they extend no further than the 1.5-fold IQR. C) Analysis of the ML predictions based on *MKI67* expression only. Left panel: Confusion matrix associated with the machine learning predictions based on *MKI67* expression. Middle panel: Kaplan-Meier curves of the overall survival of the different ML-predictions groups. The colour associated to each group matches that of the confusion matrix (left panel). Right panel: Forest plot of hazard ratios of overall survival for a model based on the ML predictions based on *MKI67* expression. For all forest plots, the black box represents estimated hazard ratios and whiskers represent the associated 95% confidence intervals. Wald test p-values are shown on the right; $0.01 \leq p < 0.05$, $0.001 \leq p < 0.01$, and $p < 0.001$ are annotated by one, two, and three stars, respectively. Number of samples (N) for each group is given in brackets. Data necessary to reproduce the figure are provided in Supplementary Data 1.

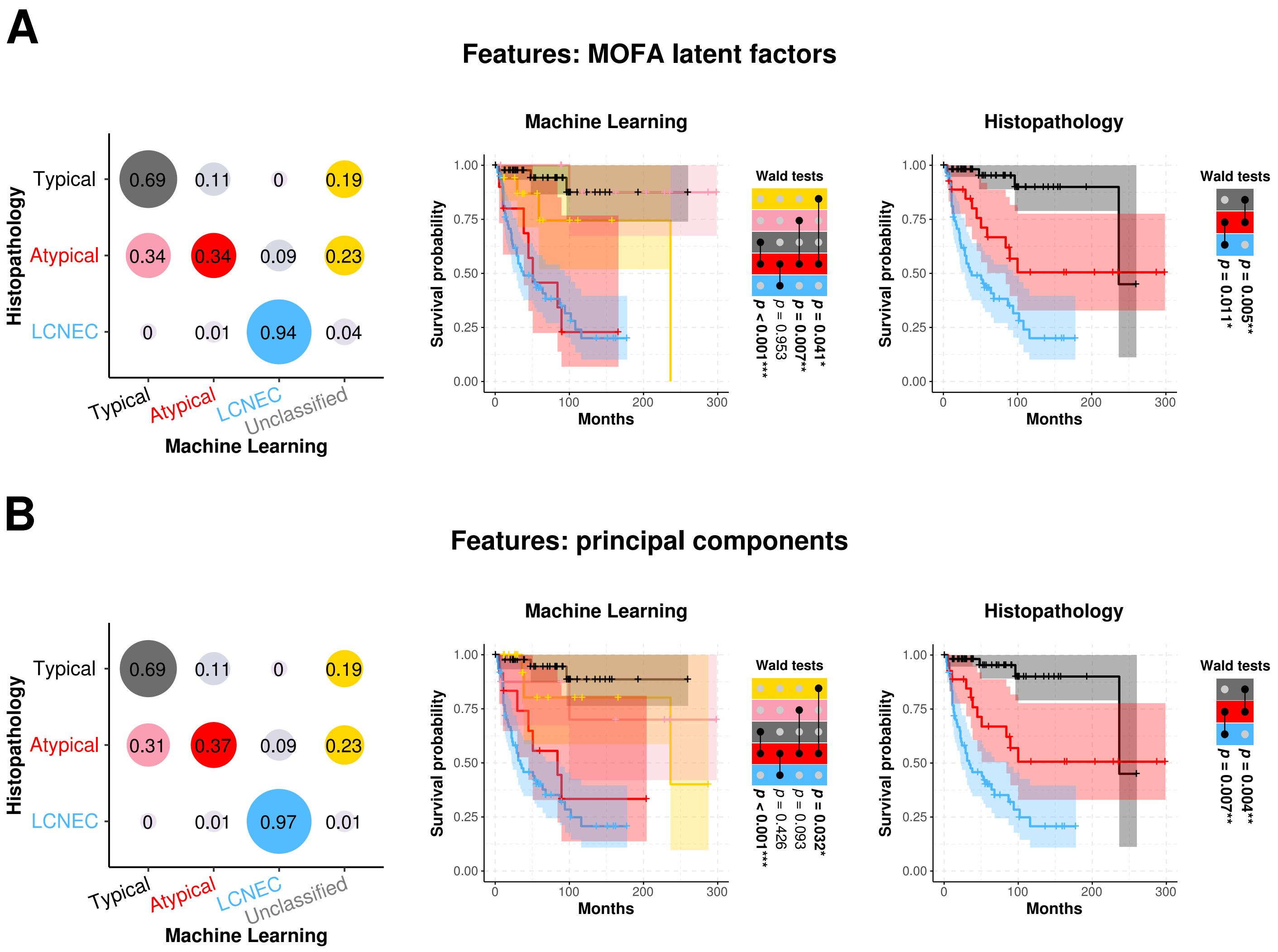

Supplementary Figure 12 Analysis of the ML predictions when considering (A) MOFA latent factors and (B) PCA principal components as features in the classification model. The analyses are similar to that used to produce Figure 1B-C, except that MOFA latent factors or PCA principal components are used instead of expression and methylation (see Online Methods). The MOFA latent factors and principal components explaining more than 2 % of the variance were used in the analysis. The design of each panel follows that of Figure 1B-C. Data necessary to reproduce the figure are provided in Supplementary Data 1.

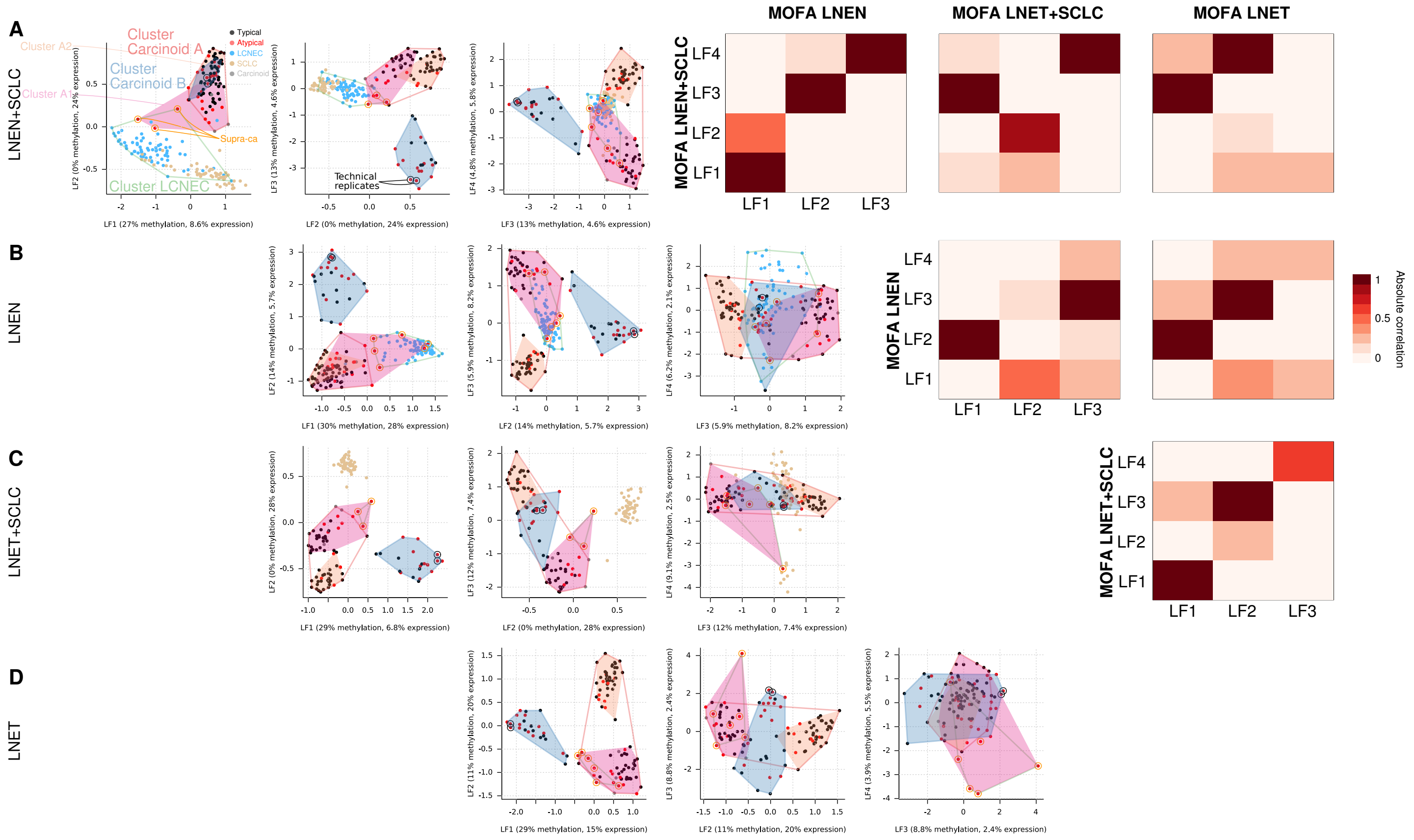

Supplementary Figure 13 Consistency of MOFA across analyses including different histopathological types. A) MOFA of transcriptomes and methylomes of LNEN and SCLC samples. B) MOFA of transcriptomes and methylomes of LNEN samples. C) MOFA of transcriptomes and methylomes of LNET and SCLC samples. D) MOFA of transcriptomes and methylomes of LNET samples. All MOFA plot designs follow that of Supplementary Figures 6 and 7. Images on the right correspond to Pearson correlation coefficients between latent factors (LF) from different analyses (e.g., MOFA LF1 from panel A with MOFA LF2 from panel B, where colors correspond to the strength of the absolute correlation; light: weak, dark brown: strong). Data necessary to reproduce the figure are provided in Supplementary Data 1.

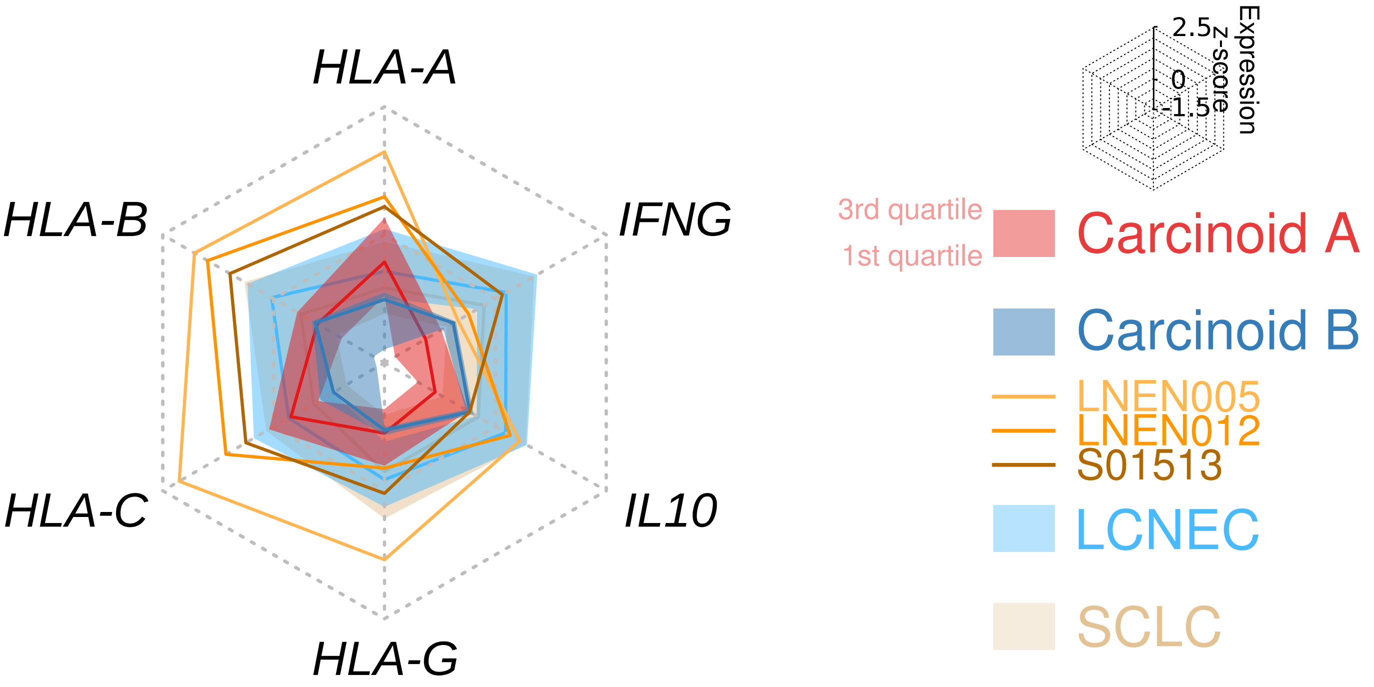

Supplementary Figure 14 Radar chart of the expression levels of HLA class I and related immunostimulatory genes as a function of their molecular group. Expression levels are expressed in *z*-score; the different groups correspond to the LNEN molecular clusters (Carcinoid A, Carcinoid B, and LCNEC clusters), supra-carcinoids (LNEN005, LNEN012, S01513), LCNEC, and SCLC. Data necessary to reproduce the figure are provided in Supplementary Data 1, and in the European Genome-phenome Archive.

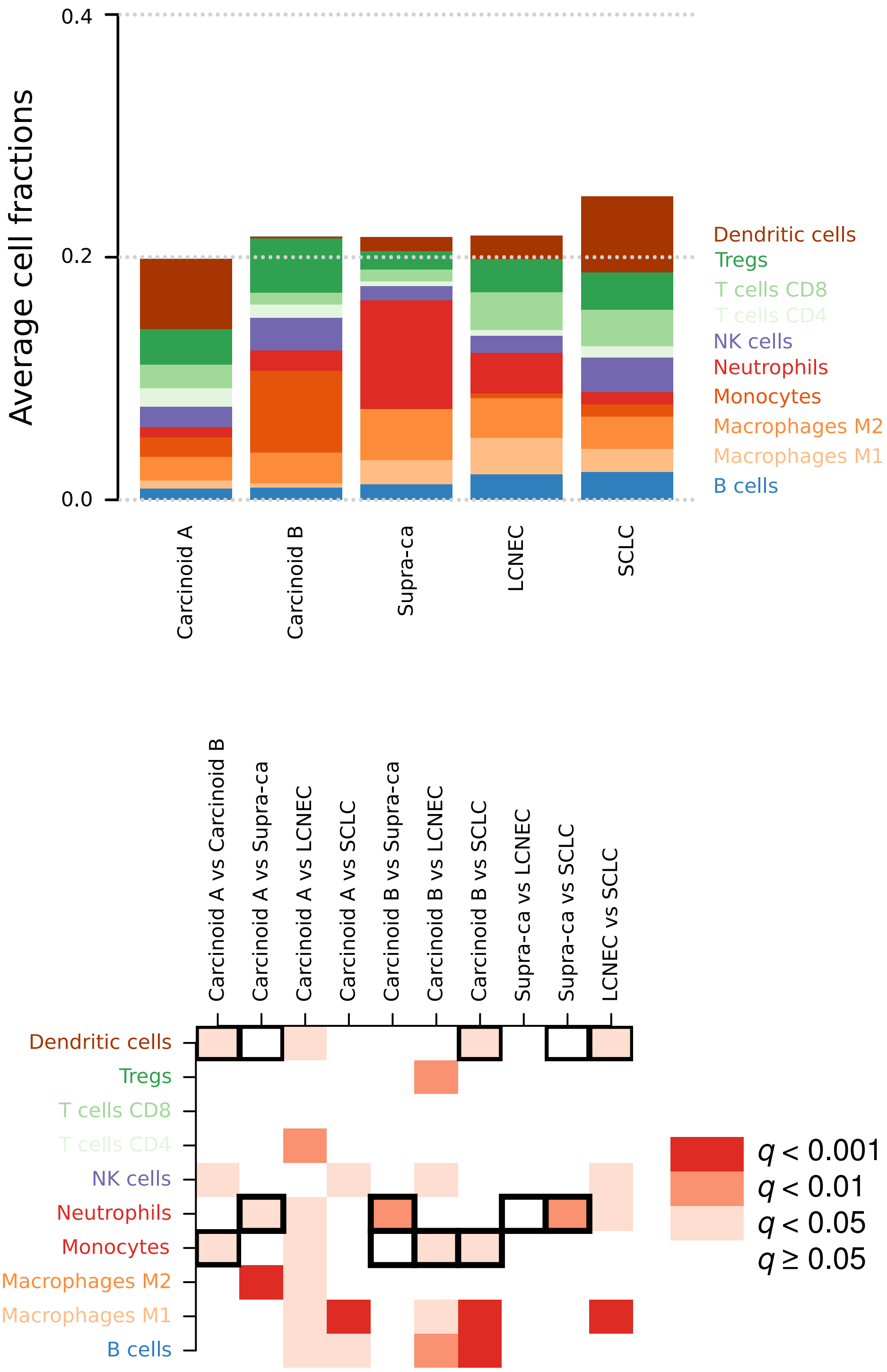

Supplementary Figure 15 Estimation of the amount of immune cells in the different pulmonary carcinoid groups from transcriptome data. The upper panel represents immune cells of each LNEN cluster and supra-carcinoids (supra-ca). The average proportion of each cell type in each group is represented. The lower panel represents the linear permutation test significance (q-value; colours: dark for $q < 0.001$, intermediate for $q < 0.01$, light for $q < 0.05$, white for $q \geq 0.05$) of the difference in cell type composition, for each cell type (row), and each possible pairwise comparison between groups (columns). Comparisons with a cell proportion difference greater than 2% are indicated by a black box. Estimates are computed using software quanTIseq (see Online methods). Data necessary to reproduce the figure are provided in Supplementary Data 1.

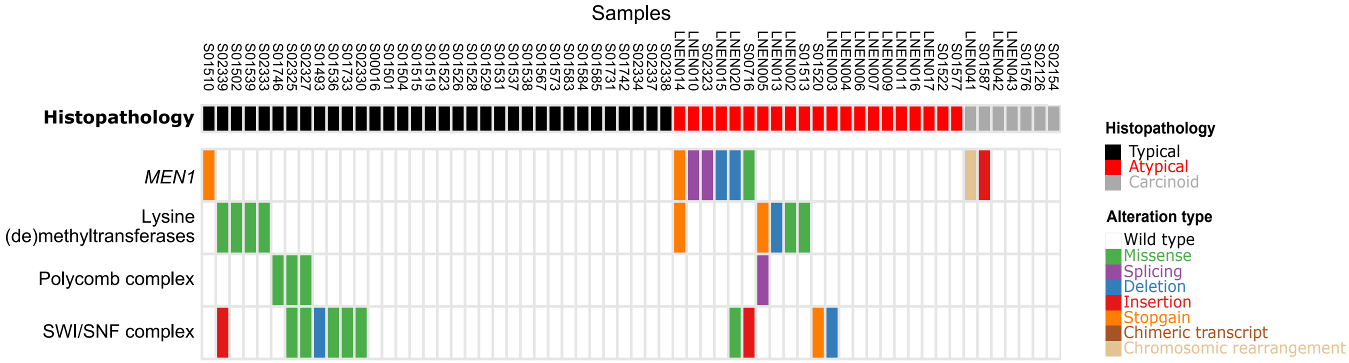

Supplementary Figure 16 Cancer-relevant somatically altered pathways altered in typical and atypical carcinoids. Colours correspond to the different types of genomic alterations. Data necessary to reproduce the figure are provided in Supplementary Data 4.

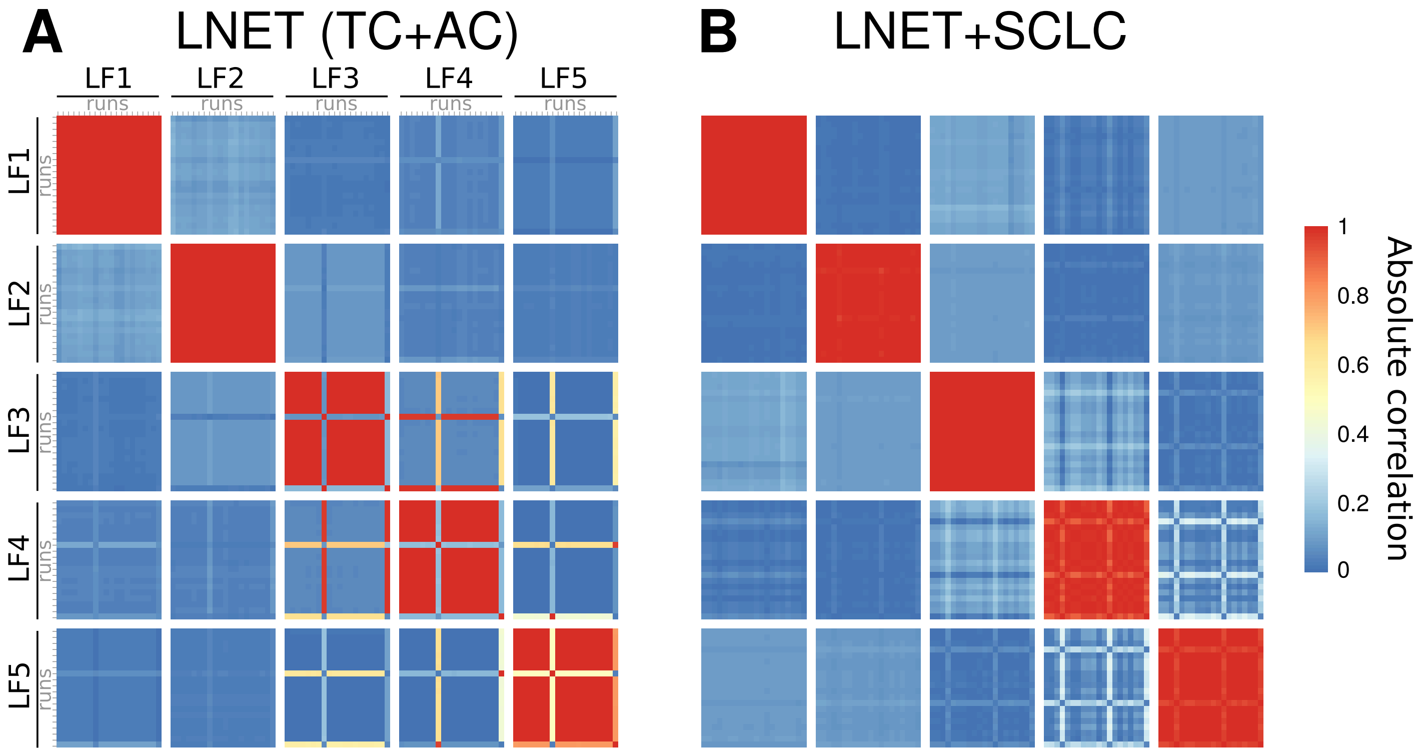

Supplementary Figure 17 Robustness of the MOFA latent factors presented in Figure 4A. A) Correlation between LF across runs for MOFA run on all LNET samples (the best run among the 20 is presented Figure 4A and Supplementary Figure 13D). B) Correlation between LF across runs for MOFA run on all LNET or SCLC samples (the best run among the 20 is presented Supplementary Figure 13C). Figure design follows that of Supplementary Figure 2. Data necessary to reproduce the figure are provided in Supplementary Data 1.

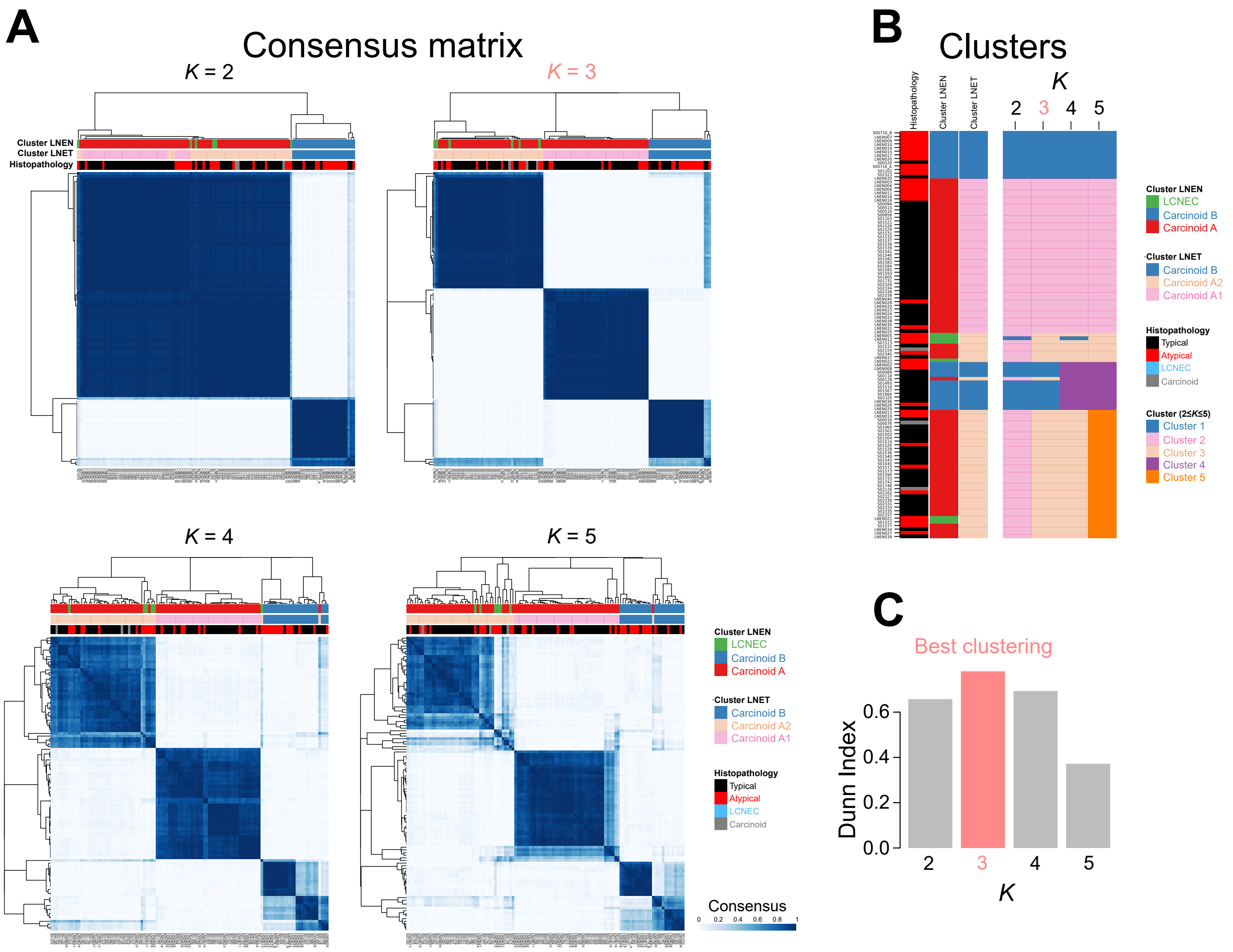

Supplementary Figure 18 Robustness of the consensus clustering of pulmonary carcinoids presented in Figure 4A. A) Heatmap of the consensus matrix for four numbers of clusters *K*; cluster memberships and histopathological types are reported above the columns, and the dendrogram represents a hierarchical clustering. B) Cluster membership as a function of *K*. C) Clustering quality metric (Dunn Index) for each value of *K*; the best clustering according to the metric is highlighted in pink. Data necessary to reproduce the figure are provided in Supplementary Data 1.

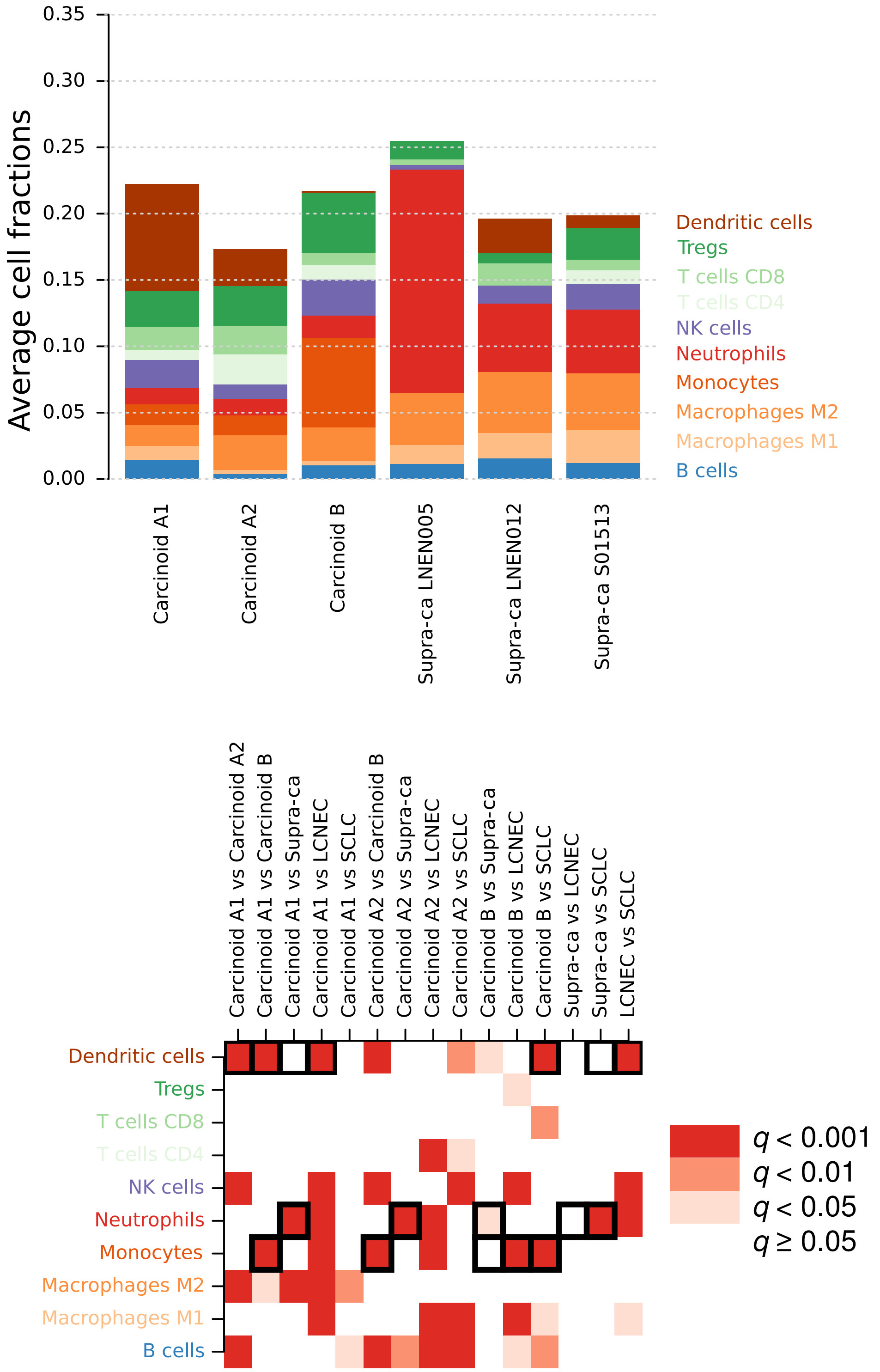

Supplementary Figure 19 Estimation of the amount of immune cells in the different LNET clusters and supra-carcinoids from transcriptome data. Figure design follows that of Supplementary Figure 15. Data necessary to reproduce the figure are provided in Supplementary Data 1.

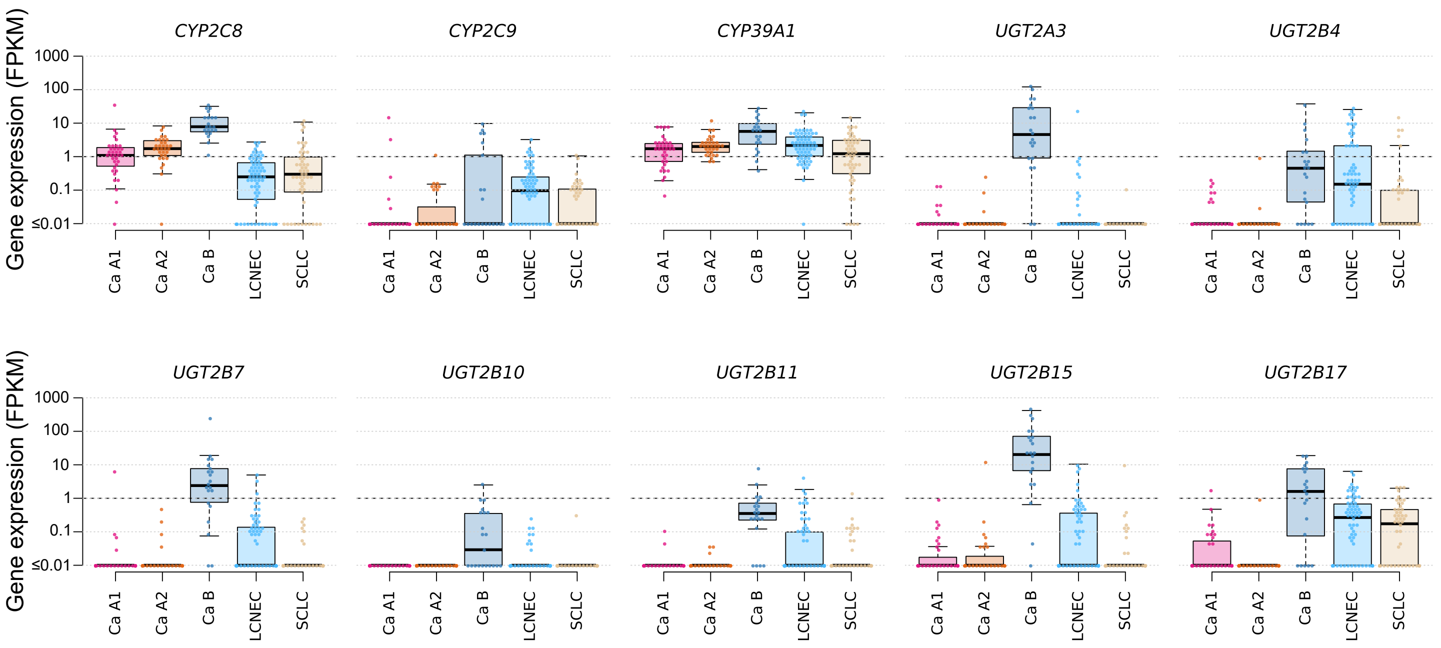

Supplementary Figure 20 Expression levels of genes involved in phase I and phase II (cytochrome P450) xenobiotic metabolism in the different LNET clusters, LCNEC and SCLC. Expression is measured in fragments per kilobase million (FPKM) units; in each plot, beeswarm plots are superimposed to boxplots to display the distribution of expression level in the corresponding groups. Centre line represents the median and box bounds represent the inter-quartile range (IQR). The whiskers span a 1.5-fold IQR or the highest and lowest observation values if they extend no further than the 1.5-fold IQR. Data necessary to reproduce the figure are provided in Supplementary Data 1, and in the European Genome-phenome Archive.

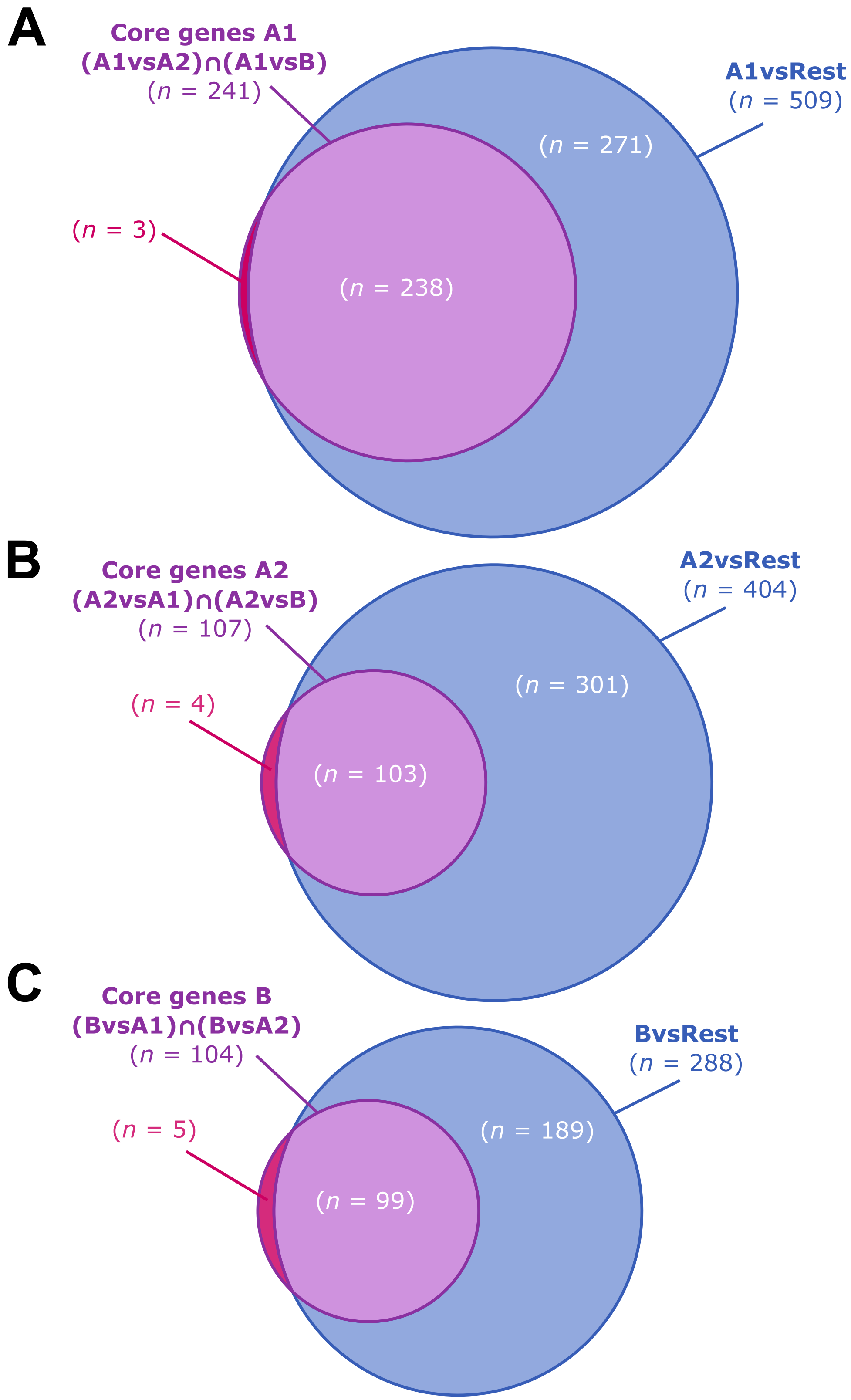

Supplementary Figure 21 Comparison of two methods to identify core differentially expressed (DE) genes of LNET clusters. Panels (A), (B), and (C) present VENN diagrams contrasting the sets of genes that are DE in all pairwise comparisons between the focal group and other groups [e.g., denoted (A1vsA2)∩(A1vsB)], and the set of genes that are DE between the focal group and all the rest (e.g., denoted A1vsRest).

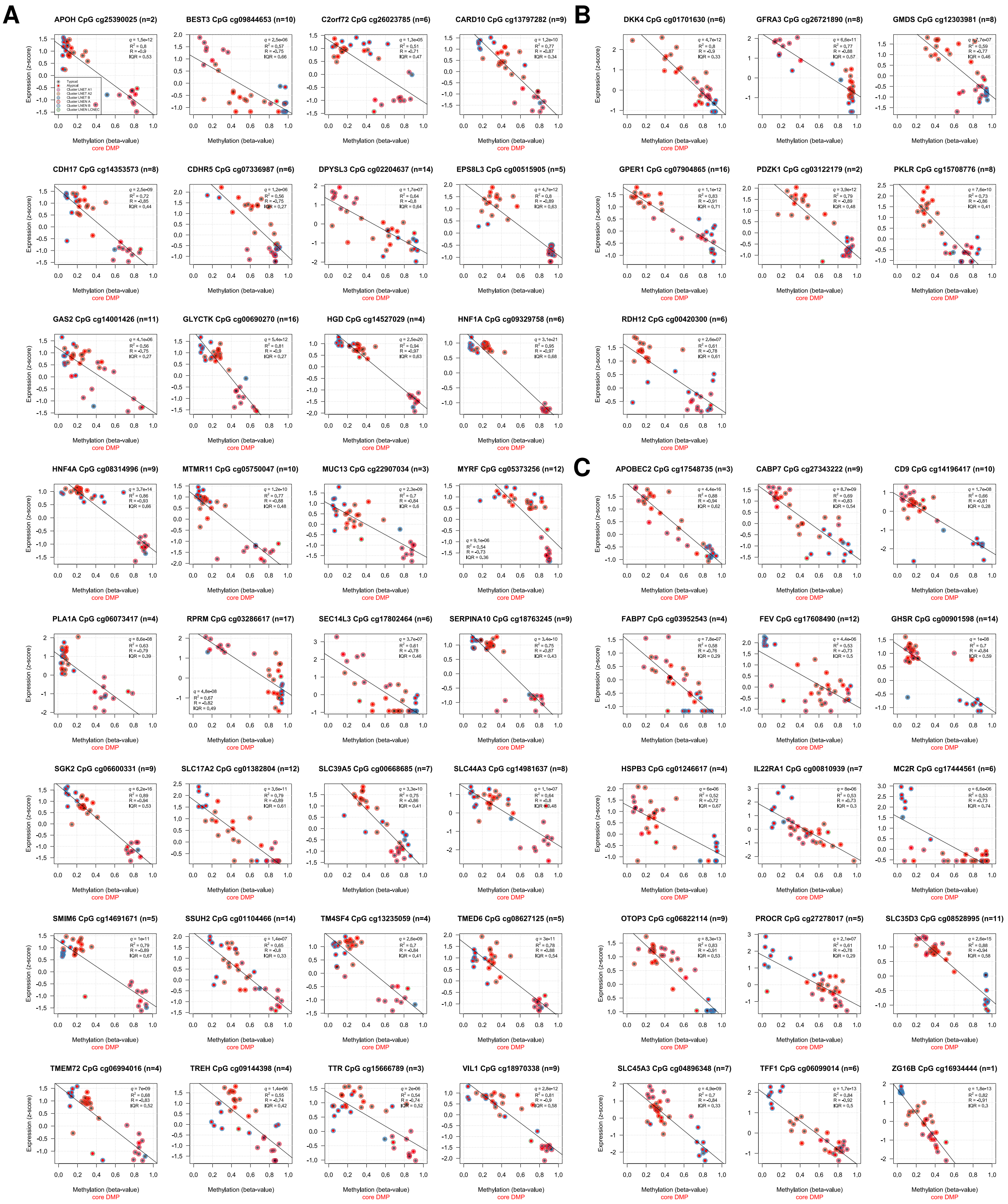

Supplementary Figure 22 Correlations between DNA methylation and gene expression for core genes of LNET clusters. Panels (A), (B), and (C) provide DNA methylation and gene expression correlations in cluster A1, A2 and B, respectively. For each coding gene, we only represent the CpGs from the promoter region and that display the strongest association (see Online Methods). Each plot represents the correlation between the β-values of the CpG and the z-scores of the corresponding gene; lines represent the best linear model fit; point colors represent the histopathological type; inner circles represent LNET clusters, outer circles represent LNEN clusters. Pearson correlation coefficients (R), corresponding correlation test q-values, and inter-quartile ranges (IQR) of the distribution of β-values of the CpG are mentioned in the top right. The number of CpGs associated with each gene, denoted by n, is mentioned in the title of each plot. If the represented CpG belongs to the core DMP of the cluster, this is mentioned in red under each plot. Data necessary to reproduce the figure are provided in Supplementary Data 10 and 11.

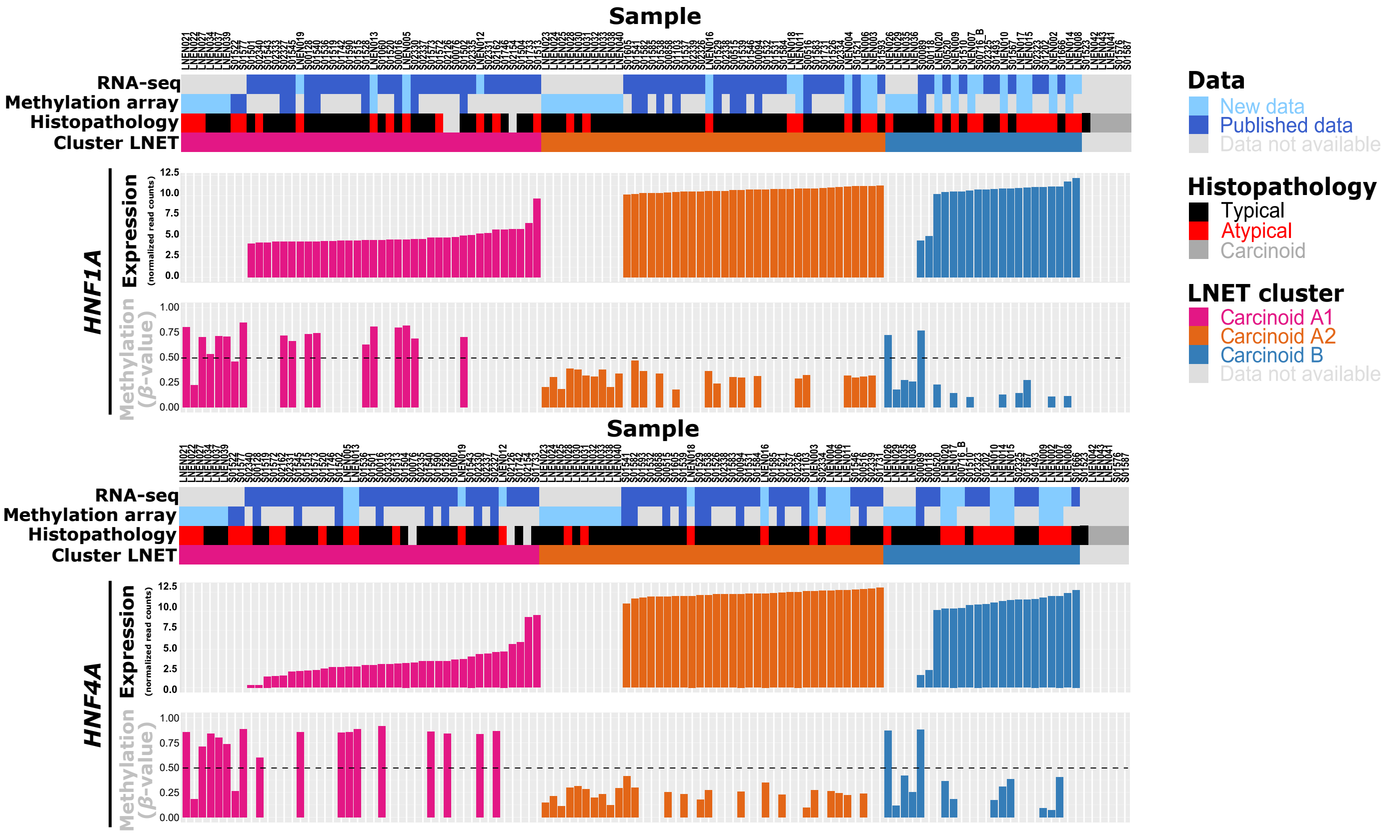

Supplementary Figure 23 DNA methylation and gene expression levels of *HNF1A* and *HNF4A* in LNET samples. DNA methylation levels correspond to the mean β-value of the CpGs correlated to the gene expression from Supplementary Data 10. Data necessary to reproduce the figure are provided in Supplementary Data 1, 10, and in the European Genome-phenome Archive.

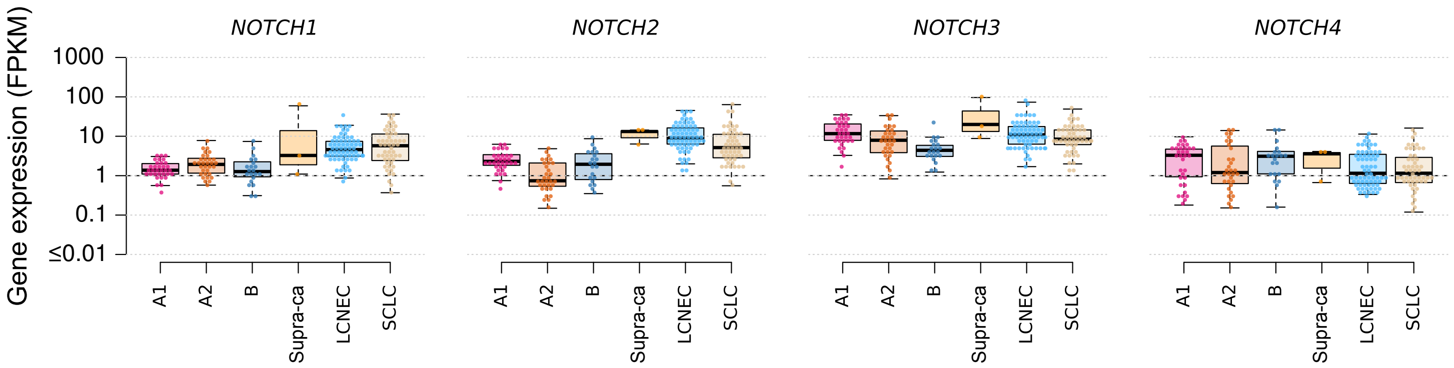

Supplementary Figure 24 Expression levels of NOTCH genes in the different LNET clusters, supra-ca, LCNEC and SCLC. The design of each panel follows that of Supplementary Figure 20. Data necessary to reproduce the figure are provided in Supplementary Data 1 and in the European Genome-phenome Archive.

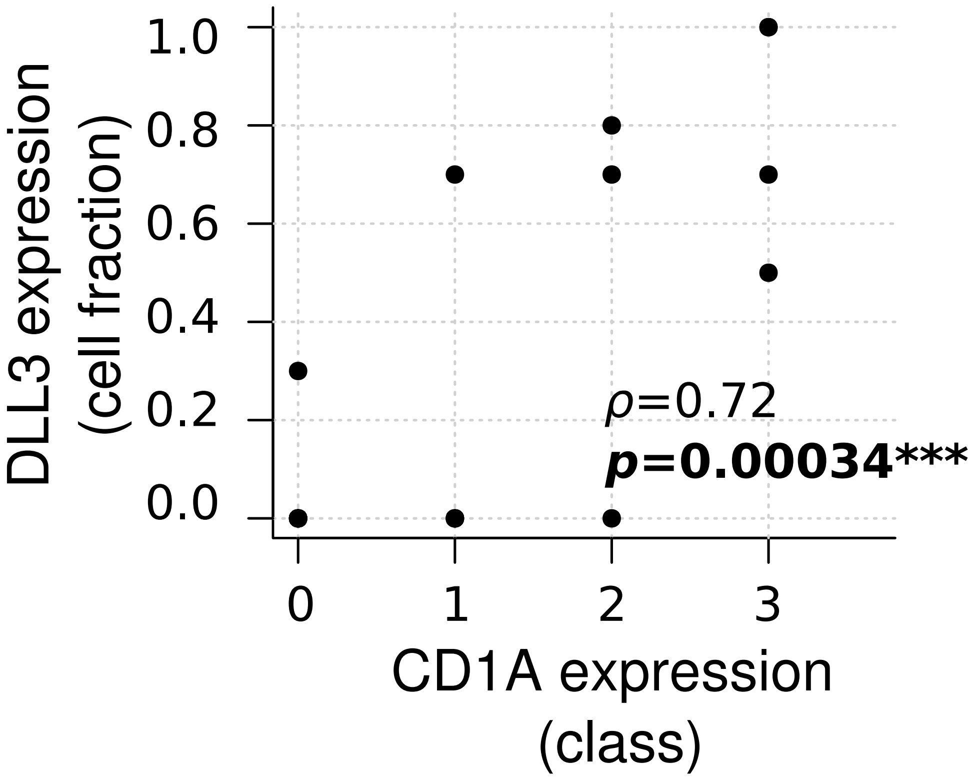

Supplementary Figure 25 Correlation between DLL3 and CDA1 expression based on immunohistochemistry in a validation series. The fraction of tumor cells exhibiting a cytoplasmic staining for DLL3 are represented on the y axis. The x axis corresponds to the CDA1 positivity classes based on the percentage of the total surface of the tumour exhibiting a membrane staining: 1 corresponds to less than 1%, 2 to a percentage between 1% and 5%, and 3 to more than 5%. The p-value and correlation coefficients of the Spearman correlation test are mentioned; $0.01 \leq p < 0.05$, $0.001 \leq p < 0.01$, and $p < 0.001$ are annotated by one, two, and three stars, respectively. Data necessary to reproduce the figure are provided in Supplementary Data 9.

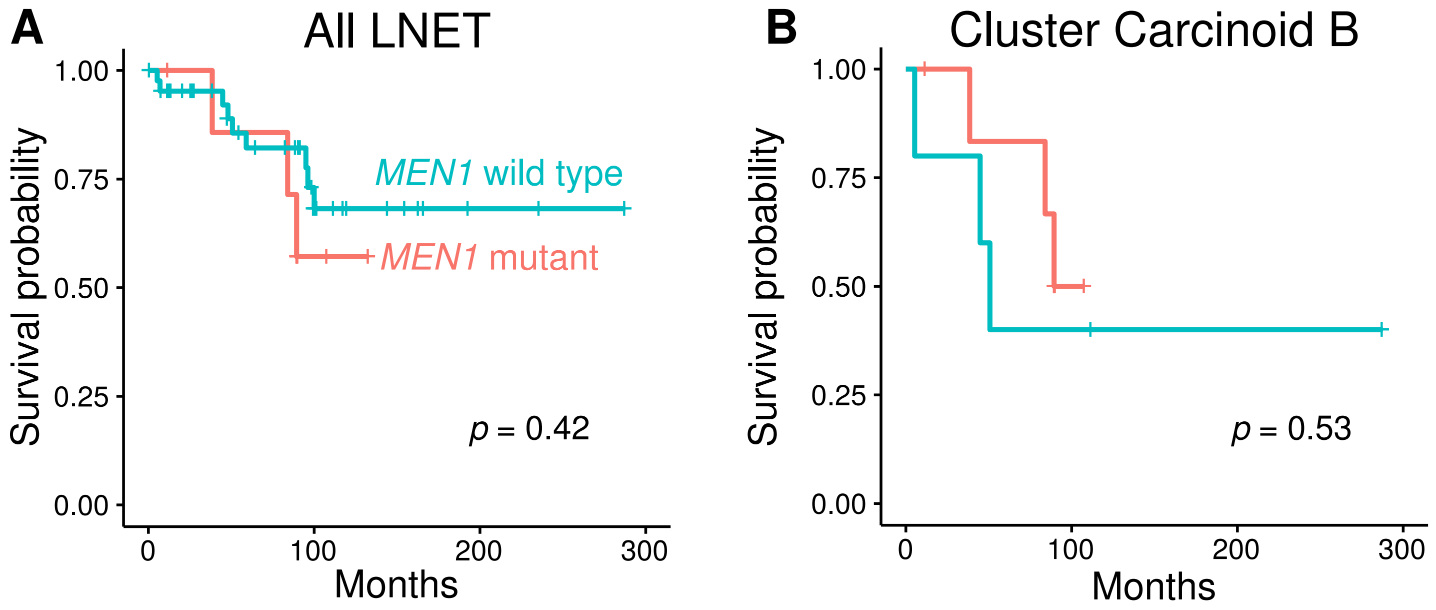

Supplementary Figure 26 Survival (Kaplan-Meier curve) of *MEN1* wild type compared to mutant cases. A) Analysis with all LNET samples. B) Analysis restricted to cluster Carcinoid B samples. The logrank test *p*-value is given at the bottom right for each panel. Data necessary to reproduce the figure are provided in Supplementary Data 1 and 4.

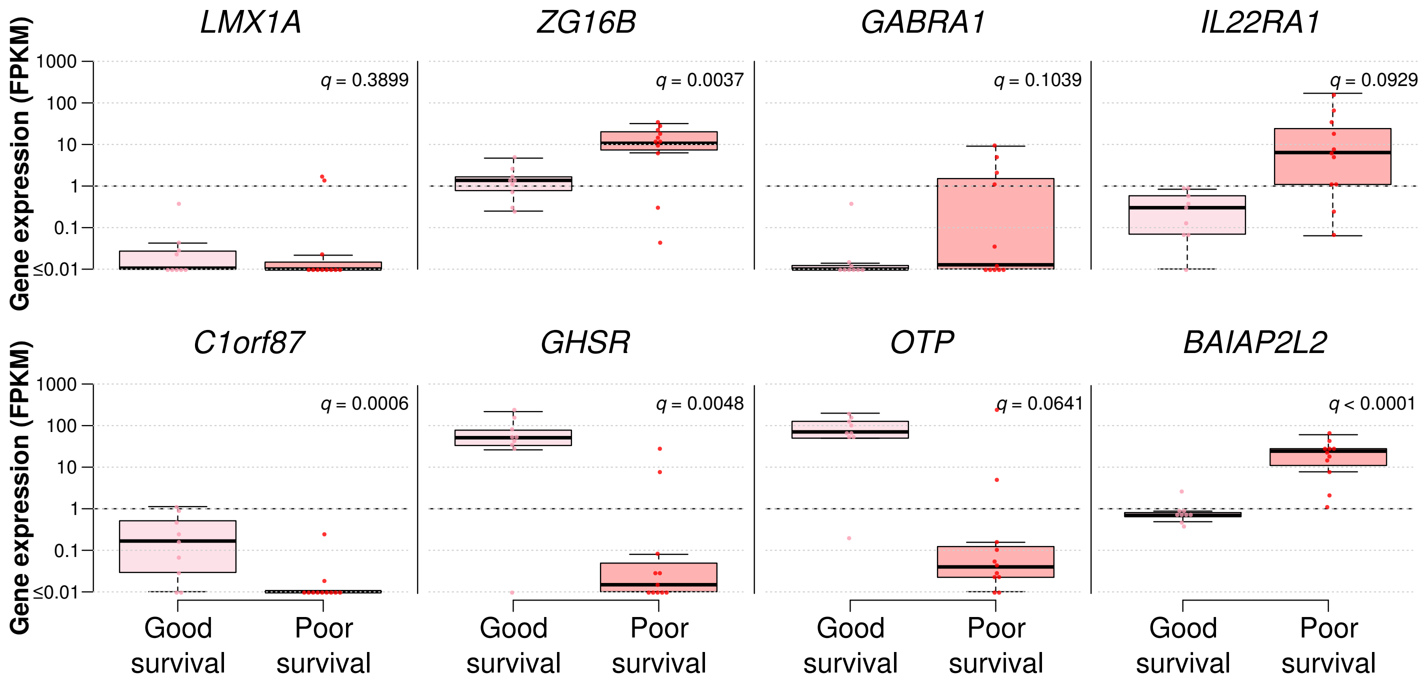

Supplementary Figure 27 Expression levels of core cluster B genes associated with survival (Figure 1B). For each gene selected by the penalized Cox regression (Supplementary Data 13), the expression levels between the good- (histopathological (HP) atypical predicted by the machine learning (ML) as typical, in pink) and poor-prognosis groups of atypical carcinoids (HP-atypical predicted as ML-atypical, in red) are compared. Expression is measured in fragments per kilobase million (FPKM) units; in each plot, beeswarm plots are superimposed to boxplots to display the distribution of expression level in the corresponding groups. Centre line represents the median and box bounds represent the inter-quartile range (IQR). The whiskers span a 1.5-fold IQR or the highest and lowest observation values if they extend no further than the 1.5-fold IQR. The q-values corresponds to the Benjamini-Hochberg adjusted p-value of permutation tests. Data necessary to reproduce the figure are provided in Supplementary Data 1, 13, and in the European Genome-phenome Archive.

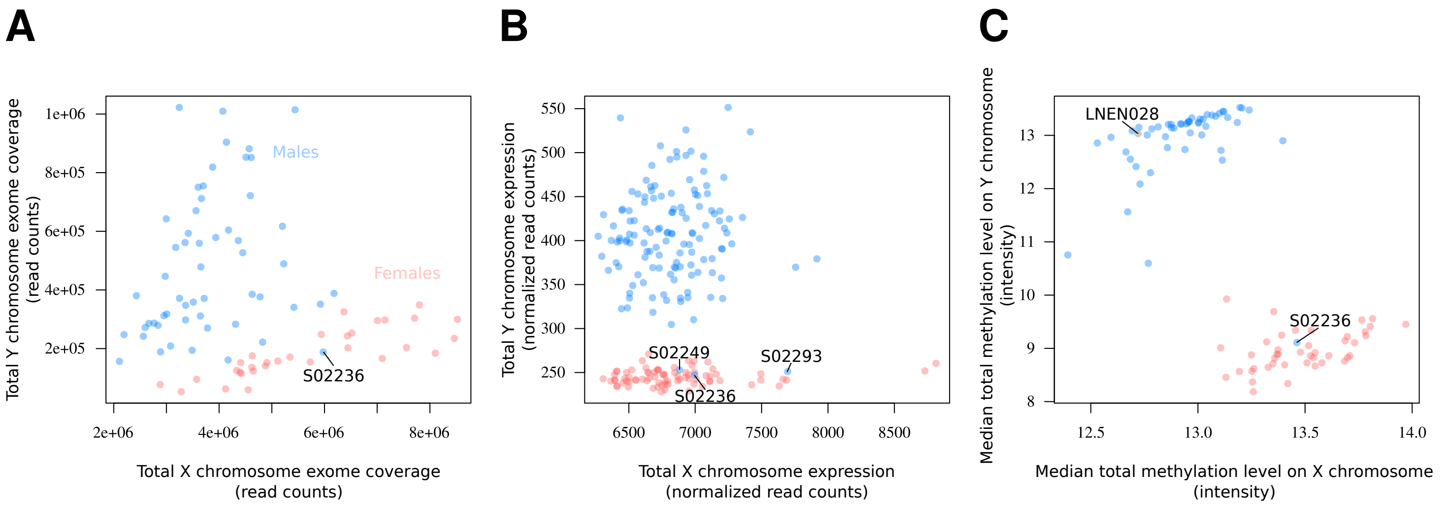

Supplementary Figure 28 Sex reclassification and multi-omic validation of reported clinical sex. A) Total exome reads coverage on the X and Y chromosomes for each sample. B) Total expression level of each sample on the X and Y chromosomes (in variance-stabilized read counts). C) Median methylation array total intensity on the X and Y chromosomes. In each panel, point colors correspond to the sexes (blue for male, red for female), and samples with discordant reported clinical sex and molecular patterns on sex chromosomes are indicated. Data necessary to reproduce the figure are provided in Supplementary Data 1, and in the European Genome-phenome Archive.

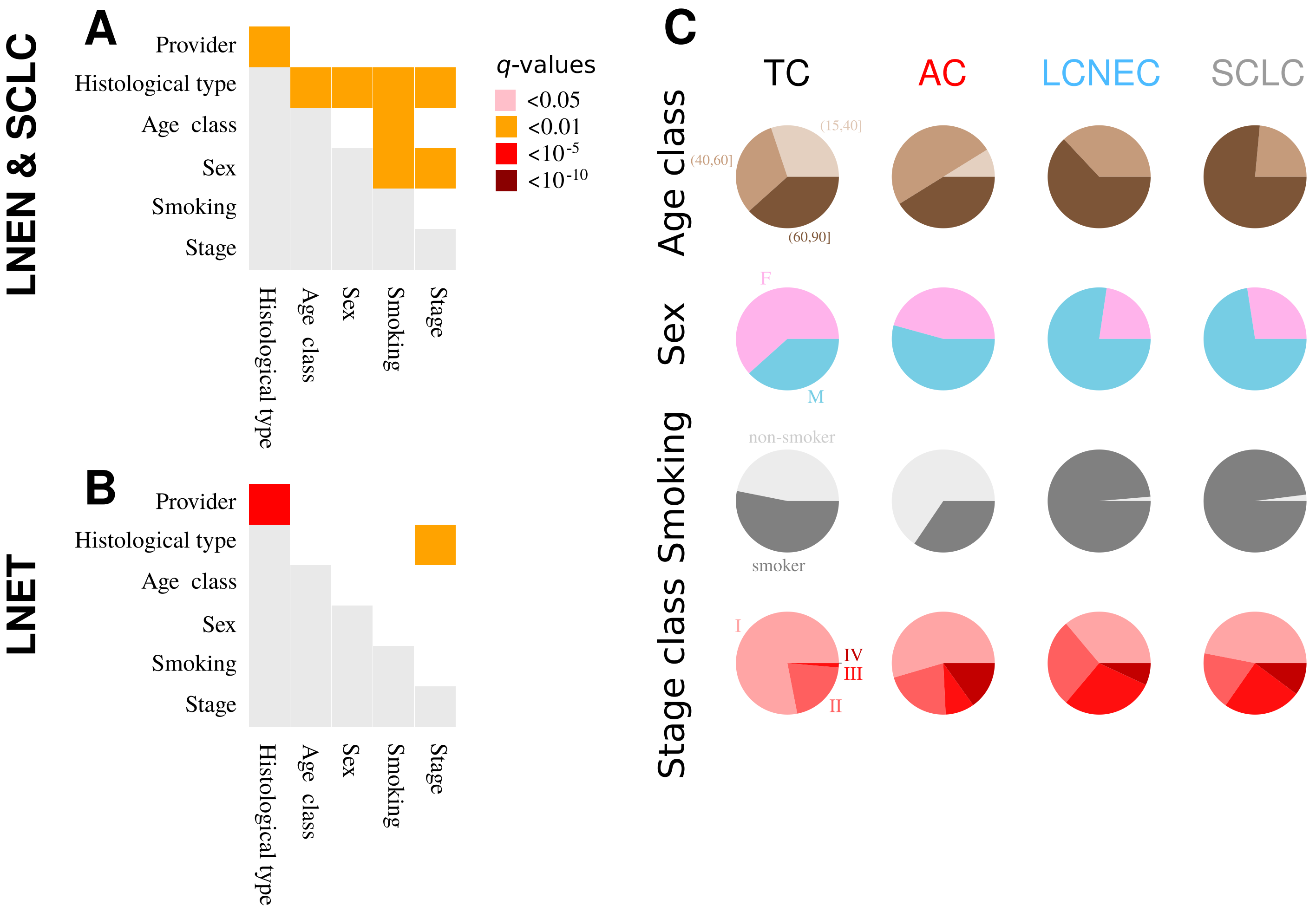

Supplementary Figure 29 Associations between clinical variables. A) Matrix of the significance (q-value) of the associations between pairs of variables, for all 242 samples from Supplementary Data 1. B) Matrix of the significance (q-value) of the association between pairs of variables, for all 116 LNET samples from Supplementary Data 1. C) Proportion of each level of each variable (rows) for each histopathological type (columns). In (A) and (B), associations are computed using Fishers exact test, adjusting for multiple testing using the Benjamini-Hochberg procedure; because of symmetry, only the upper diagonal was tested and represented. Data necessary to reproduce the figure are provided in Supplementary Data 1.

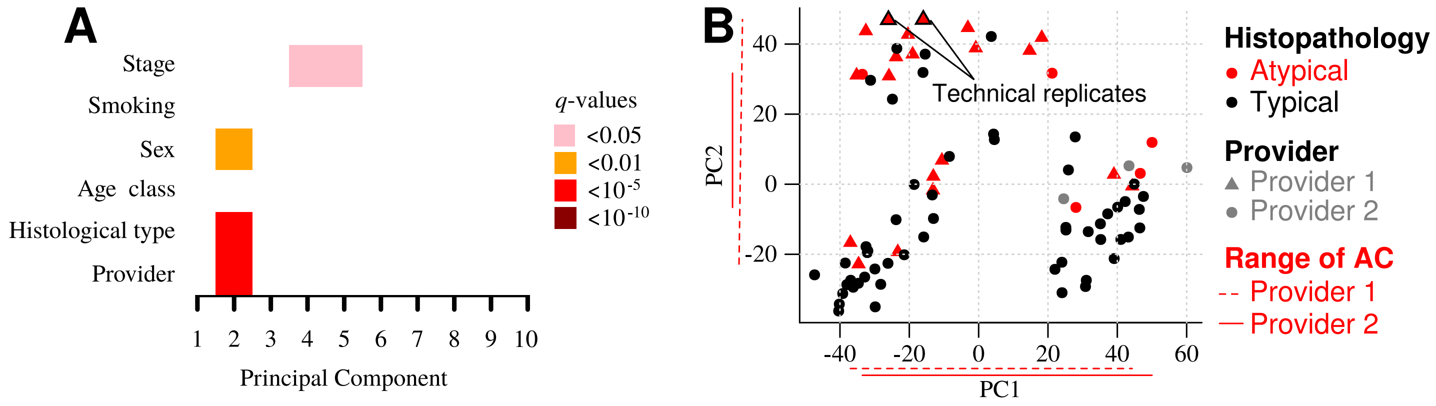

Supplementary Figure 30 Associations between clinical variables and expression profiles of LNET. A) Matrix of the significance (q-value) of the associations, computed using Fishers exact test, between clinical variables and expression principal components. B) First two axes of the PCA from panel A, with sample providers highlighted (point shapes); red segments next to the axes indicate the range of the distribution of atypical carcinoids (AC) from each provider on each principal component. Figure design follows that of Supplementary Figure 29. Data necessary to reproduce the figure are provided in Supplementary Data 1.

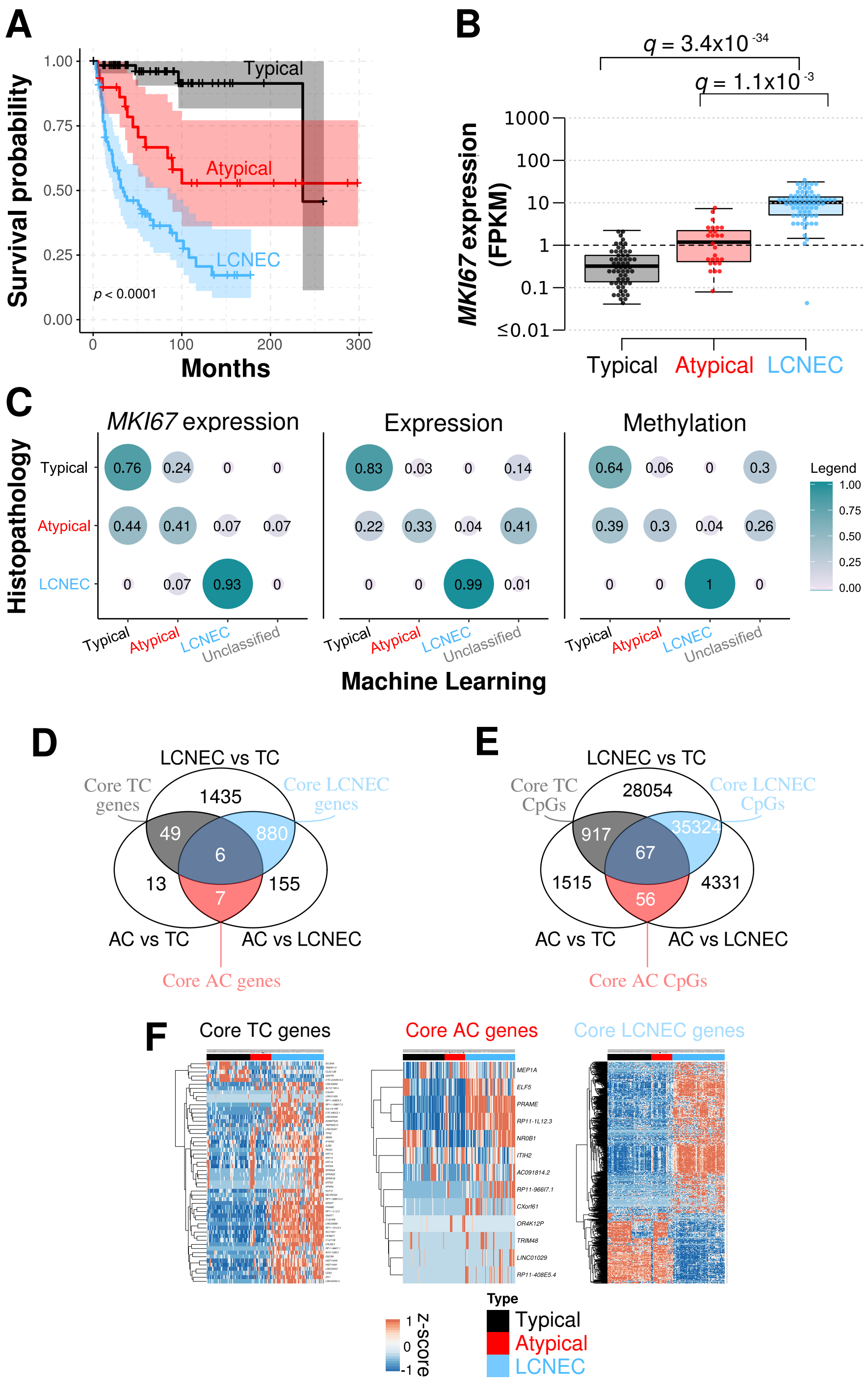

Supplementary Figure 31 Supervised analysis of histological types. A) Kaplan-Meier curve of overall survival of histopathological types (logrank test *p*-value is given bottom left). B) Boxplot of the expression level (in Fragments Per Kilobase Million; FPKM) of *MKI67* for each histopathological type. Centre line represents the median and box bounds represent the inter-quartile range (IQR). The whiskers span a 1.5-fold IQR or the highest and lowest observation values if they extend no further than 1.5-fold IQR. The differential expression analysis *q*-value obtained from transcriptome-wide comparisons (Supplementary Data 15) is given above each comparison. C) Machine learning analysis associated with the classification of typical carcinoids, atypical carcinoids, and LCNEC. Left panel: confusion matrix associated with the classification based on *MKI67* expression only. Middle panel: confusion matrix associated with the classification based on expression data. Right panel: confusion matrix associated with the classification based on methylation data. D) Venn diagram of core differentially expressed genes in pairwise comparisons between histopathological types. E) Venn diagram of core CpGs in pairwise comparisons between histopathological types. F) Expression of core differentially expressed genes for each histopathological type. Data necessary to reproduce the figure are provided in Supplementary Data 1, 15, and in the European Genome-phenome Archive.

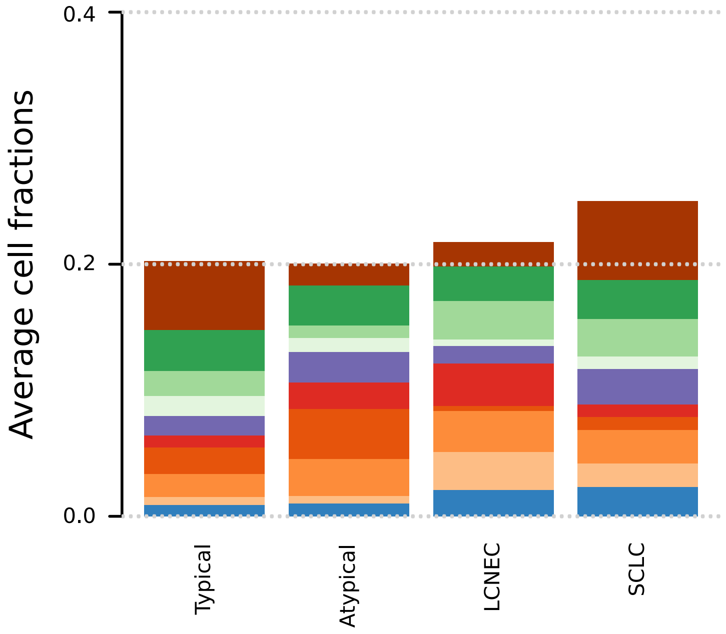

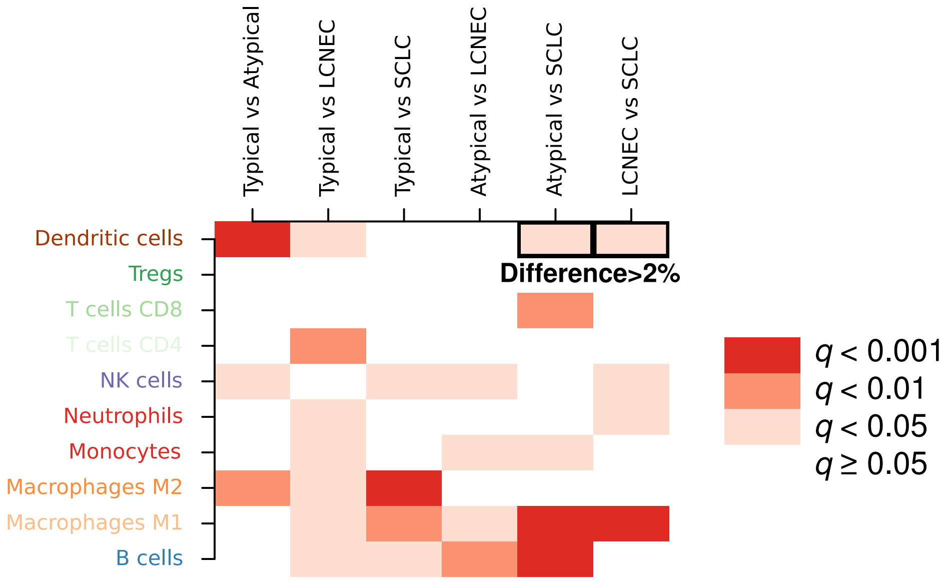

Supplementary Figure 32 Estimation of the amount of immune cells in the different histopathological types from transcriptome data. Figure design follows that of Supplementary Figures 15 and 19. Data necessary to reproduce the figure are provided in Supplementary Data 1.

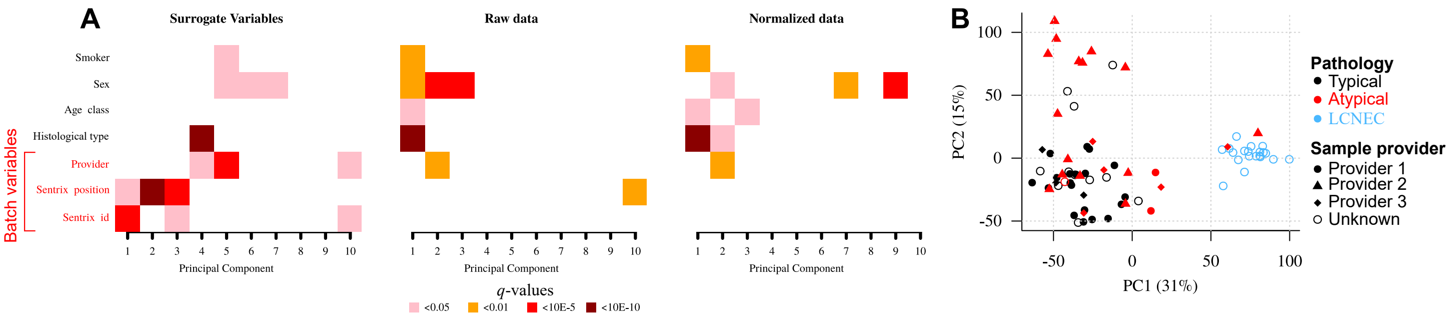

Supplementary Figure 33 Assessment of the batch effects in the EPIC 850K methylation array analysis. A) Matrix of the significance (q-value) of the associations, computed using Fishers exact test, between batch and clinical variables and: i) methylation surrogate variables determined from non-negative control probes (left panel), ii) the principal components of the most variable M-values (Online Methods), before functional normalization (middle panel), iii) the principal components of the most variable M-values (Online Methods), after functional normalization (right panel). B) First two axes of the PCA from panel C, with sample providers and histolopathological types highlighted (point shapes and colors, respectively). Figure design follows that of Supplementary Figures 29 and 30. Data necessary to reproduce the figure are provided in Supplementary Data 1.
