# Supplementary material for: Integrative and comparative genomic analyses identify clinically relevant pulmonary carcinoid groups and unveil the supra-carcinoids
Source: Nat Commun. 2019 Aug 20;10:3407. doi: 10.1038/s41467-019-11276-9 (PMC6702229; doi:10.1038/s41467-019-11276-9)
Supplement: Supplementary file 3 — Description of Additional Supplementary Files [file 41467_2019_11276_MOESM3_ESM.pdf]

Description of Additional Supplementary Files

File Name: Supplementary Data 1
Description: Sample overview

File Name: Supplementary Data 2
Description: Principal Components based on RNA-seq data

File Name: Supplementary Data 3
Description: Principal Components based on methylation data

File Name: Supplementary Data 4
Description: Somatic mutations

File Name: Supplementary Data 5
Description: Hallmarks of cancer gene set enrichment analysis

File Name: Supplementary Data 6
Description: Gene set enrichment analysis for MOFA latent factors

File Name: Supplementary Data 7
Description: Chimeric transcripts

File Name: Supplementary Data 8
Description: Chromosomic rearrangements

File Name: Supplementary Data 9
Description: DLL3 and CD1A Immunohistochemistry

File Name: Supplementary Data 10
Description: Differentially expressed genes between MOFA LNET clusters

File Name: Supplementary Data 11
Description: Differentially methylated positions between MOFA LNET clusters.

File Name: Supplementary Data 12
Description: Differentially expressed genes between MOFA LNEN clusters

File Name: Supplementary Data 13
Description: Genes selected by the regularized GLM model based on the expression of core genes of LNET cluster B

File Name: Supplementary Data 14
Description: Summary statistics

File Name: Supplementary Data 15
Description: Differentially expressed genes between histological types

File Name: Supplementary Data 16
Description: Differentially methylated positions between histological types

File Name: Supplementary Data 17
Description: Differentially methylated positions between MOFA LNEN clusters

File Name: Supplementary Data 18
Description: Software summary

File Name: Supplementary Software 1
Description: R scripts used for data analysis
