# Supplementary material for: Integrative and comparative genomic analyses identify clinically relevant pulmonary carcinoid groups and unveil the supra-carcinoids
Source: Nat Commun. 2019 Aug 20;10:3407. doi: 10.1038/s41467-019-11276-9 (PMC6702229; doi:10.1038/s41467-019-11276-9)
Supplement: Supplementary file 23 — Reporting Summary [file 41467_2019_11276_MOESM23_ESM.pdf]

nature research

Corresponding author(s): Lynnette Fernandez-Cuesta

Last updated by author(s): May 15, 2019

Reporting Summary

Nature Research wishes to improve the reproducibility of the work that we publish. This form provides structure for consistency and transparency in reporting. For further information on Nature Research policies, see Authors & Referees and the Editorial Policy Checklist.

Statistics

For all statistical analyses, confirm that the following items are present in the figure legend, table legend, main text, or Methods section.

n/a	Confirmed	
☐	☒	The exact sample size (*n*) for each experimental group/condition, given as a discrete number and unit of measurement
☐	☒	A statement on whether measurements were taken from distinct samples or whether the same sample was measured repeatedly
☐	☒	The statistical test(s) used AND whether they are one- or two-sided<br>*Only common tests should be described solely by name; describe more complex techniques in the Methods section.*
☐	☒	A description of all covariates tested
☐	☒	A description of any assumptions or corrections, such as tests of normality and adjustment for multiple comparisons
☐	☒	A full description of the statistical parameters including central tendency (e.g. means) or other basic estimates (e.g. regression coefficient) AND variation (e.g. standard deviation) or associated estimates of uncertainty (e.g. confidence intervals)
☐	☒	For null hypothesis testing, the test statistic (e.g. *F*, *t*, *r*) with confidence intervals, effect sizes, degrees of freedom and *P* value noted<br>*Give P values as exact values whenever suitable.*
☒	☐	For Bayesian analysis, information on the choice of priors and Markov chain Monte Carlo settings
☒	☐	For hierarchical and complex designs, identification of the appropriate level for tests and full reporting of outcomes
☐	☒	Estimates of effect sizes (e.g. Cohen's *d*, Pearson's *r*), indicating how they were calculated

Our web collection on statistics for biologists contains articles on many of the points above.

Software and code

Policy information about availability of computer code

Data collection	No software was used for data collection.
Data analysis	WGS and WES reads mapping: in-house workflow (github.com/IARCbioinfo/alignment-nf revision 9092214665) that used bwa version 0.7.12-r1044, samblaster version 0.1.22, sambamba version 0.5.9. Variant calling: Needlestack version 1.1, ANNOVAR version of April 16 2018. Targeted sequencing reads processing: Torrent Suite software version 4.4.2, ABRA version 0.97bLE. Copy number variant calling: WGinR (github.com/aviari/wginr version 1b6bdb1). Structural variant calling: crisscross (github.com/anso-sertier/crisscross version dcdb5e4). RNA-seq data processing: in-house workflow (github.com/IARCbioinfo/RNAseq-nf revision da7240d) that used Trim Galore version 0.4.2, STAR version 2.5.2b, htseq version 0.8.0, StringTie version 1.3.3b. Quality control with FastQC version 0.11.5, RSeQC version 2.6.4, MultiQC version 0.9. Fusion transcript discovery: TRUP (github.com/ruping/TRUP version 08ec4f4). Immune contexture quantification: quanTIseq version of March 2018. Statistical analyses with R version 3.4.1.

For manuscripts utilizing custom algorithms or software that are central to the research but not yet described in published literature, software must be made available to editors/reviewers. We strongly encourage code deposition in a community repository (e.g. GitHub). See the Nature Research guidelines for submitting code & software for further information.

Data

Policy information about availability of data

All manuscripts must include a data availability statement. This statement should provide the following information, where applicable:

- Accession codes, unique identifiers, or web links for publicly available datasets
- A list of figures that have associated raw data
- A description of any restrictions on data availability

The exome sequencing data, RNA-seq data, and methylation data have been deposited in the the European Genome-phenome Archive (EGA) database which is hosted at the EBI and the CRG, under accession number EGAS#1096. Other datasets referenced during the study are available from the EGA website. All the other

data supporting the findings of this study are available within the article and its supplementary information files and from the corresponding author upon reasonable request. A reporting summary for this article is available as a Supplementary Information file.

Field-specific reporting

Please select the one below that is the best fit for your research. If you are not sure, read the appropriate sections before making your selection.

☒ Life sciences ☐ Behavioural & social sciences ☐ Ecological, evolutionary & environmental sciences

For a reference copy of the document with all sections, see nature.com/documents/nr-reporting-summary-flat.pdf

Life sciences study design

All studies must disclose on these points even when the disclosure is negative.

Sample size	This study first includes a hypothesis generating, unsupervised analysis of exome, RNA-seq, and methylation data using group factor analysis (software MOFA). For such multivariate analyses, no simple power calculations are available, but recommendations for stable latent factors and weights such that the results from the sample can accurately be generalized to the population suggest n>100 (Saccenti and Timmerman J. Proteom. Res. 2016), which is in line with the sample size in our study (total of n=257 lung neuroendocrine neoplasms). Second, our study includes differential expression analyses. We computed the sample size required using the online tool RNAseqPS (Guo et al. Cancer Inform, 2014), finding that a sample size of n=48 samples per group was sufficient to reach a power of 0.8 for a false discovery rate of 0.05 and otherwise default parameters, which was in line with the sample size in our study (assuming 3 molecular clusters among the 154 samples with RNAseq, the average cluster size is 51). Third, our study includes differential methylation analyses. Using the power calculator for Illumina EPIC methylation array from Mansell et al. 2018, we determined that with 75 samples were enough to achieve a 80% power to detect CpG sites with 10% differences between two groups in more than 90% of the probes, using the recommended threshold P < 9.42x10-8. Finally, for the replication of our results, we conducted protein expression quantification through immunohistochemistry in an independent cohort. We estimated the necessary sample size using a binomial distribution fitted to our discovery cohort: given a proportion of 44/111=40% carcinoid samples in the cluster with high DLL3 and CD1a expression (cluster A1), and a power of 0.95 to detect at least 3 samples with DLL3 or CD1a expression, the required sample size is n=15, which is below the sample size that we chose (n=20).
Data exclusions	We pre-established to exclude samples from metastases rather than the primary tumor. One such sample was excluded from the study.
Replication	We conducted protein expression quantification through immunohistochemistry in an independent cohort to replicate the findings of groups of pulmonary carcinoids with DLL3 or CD1a expression and could replicate our initial findings.
Randomization	Samples were assigned a histopathological class through central pathological review. We assessed the importance of covariables like age, sex, smoking status in multivariate analyses using regression analyses. We controlled for these variables in statistical analyses by adding them as covariables in the regressions.
Blinding	The investigators were blinded to the groups when fitting the unsupervised analyses models (MOFA), because the groups emerged from these analyses. The investigators were not blinded to the histopathological class during the unsupervised analyses, but this was unnecessary because these analyses do not take into account any sample information except the mutations, gene expression, and methylation levels. The investigators could not be blinded to the subsequent differential expression and methylation analyses, which required knowledge of the group membership for each sample.

Reporting for specific materials, systems and methods

We require information from authors about some types of materials, experimental systems and methods used in many studies. Here, indicate whether each material, system or method listed is relevant to your study. If you are not sure if a list item applies to your research, read the appropriate section before selecting a response.

Materials & experimental systems

n/a	Involved in the study	
☐	☒	Antibodies
☒	☐	Eukaryotic cell lines
☒	☐	Palaeontology
☒	☐	Animals and other organisms
☐	☒	Human research participants
☒	☐	Clinical data

Methods

n/a	Involved in the study	
☒	☐	ChIP-seq
☒	☐	Flow cytometry
☒	☐	MRI-based neuroimaging

Antibodies

Antibodies used	Ventana DLL3 (clone SP347) assay, Roche Tissue Diagnostics catalog number 790-7016, lot E16785. Sigma Aldrich CD1 rabbit monoclonal antibody (clone EP3622), Roche Tissue Diagnostics catalog number 760-4525, lot V0001132.
Validation	For both techniques, a three stage indirect immunoperoxidase technique was performed on Ventana BenchMark ULTRA

automated staining module (Tucson, AZ USA) which enables a standardization of reaction time and temperature, washing procedures and development of staining and amplification. DLL3 and CD1a prediluted antibodies were used as recommended by the manufacturer (for DLL3: CC1 80 min at 100°C pre-treatment, incubation 32 min at 36°C, OptiView DAB IHC Detection Kit (Ventana Medical Systems, Roche Tissue Diagnostics; cat nb 760-700): for CD1a: CC1 pre-treatment 64 min at 95°C, incubation 32 min at 37°C, UltraView Universal DAB Detection Kit (Ventana Medical Systems, Roche Tissue Diagnostics; cat nb 760-500) and Amplification Kit (Ventana Medical Systems – Roche Tissue Diagnostics; cat nb 760-080). Negative control consisted in omission of the primary antibody and incubation with immunoglobulins of the same species.

Human research participants

Policy information about studies involving human research participants

Population characteristics	Age: for pulmonary carcinoids, median of 57 and range of [16-83]; for LCNEC, median of 64 and range of [45-90]<br>Sex: for pulmonary carcinoids, 78 females and 104 males; for LCNEC, 16 females and 58 males<br>All chemonaive at moment of sample collection.
Recruitment	There has not been a prospective recruitment of patients specifically done for this study. The biological specimens were previously collected for clinical routine and stored in the biobanks of the collaborative hospitals, which made the de-identified samples available for research.
Ethics oversight	International Agency for Research on Cancer ethics committee

Note that full information on the approval of the study protocol must also be provided in the manuscript.
